# Supplementary material for: Improving sleep and learning in rehabilitation after stroke, part 2 (INSPIRES2): study protocol for a home-based randomised control trial of digital cognitive behavioural therapy (dCBT) for insomnia
Source: BMJ Open. 2023 Apr 6;13(4):e071764. doi: 10.1136/bmjopen-2023-071764 (PMC10083871; doi:10.1136/bmjopen-2023-071764)
Supplement: Supplementary data [file bmjopen-2023-071764supp002.pdf]

Dr Melanie Fleming
Sleep-win@ndcn.ox.ac.uk

Participant identification number:

CONSENT FORM

Study Title: INSPIRES-2

If you agree, please initial box

1. I confirm that I have read the information sheet dated................... (version............) for this study. I have had the opportunity to consider the information, ask questions and have had these answered satisfactorily.		
2. I understand that my participation is voluntary and that I am free to withdraw at any time without giving any reason, without my medical care or legal rights being affected.		
3. I understand that relevant sections of my medical notes and data collected during the study may be looked at by individuals from University of Oxford, from regulatory authorities and from the NHS Trust(s), where it is relevant to my taking part in this research. I give permission for these individuals to have access to my records.		
4. I agree to take part in this study.		
Additional:		
5. *I agree to be contacted about ethically approved research studies for which I may be suitable. I understand that agreeing to be contacted does not oblige me to participate in any further studies.*	Yes	No
6. *I would like the researchers to send a letter to my GP to let them know that I am taking part in this research (if yes, please give details)*	Yes	No

Weightman M, *et al. BMJ Open* 2023; 13:e071764. doi: 10.1136/bmjopen-2023-071764

______________________ ______________________

Name of Participant *Date*

When completed: 1 for participant; 1 for researcher

If you agreed for researchers to send a letter to your GP, please provide your GP details

GP name (if known):

Surgery name:

Surgery address:

Weightman M, *et al. BMJ Open* 2023; 13:e071764. doi: 10.1136/bmjopen-2023-071764
