# Supplementary material for: Improving sleep and learning in rehabilitation after stroke, part 2 (INSPIRES2): study protocol for a home-based randomised control trial of digital cognitive behavioural therapy (dCBT) for insomnia
Source: BMJ Open. 2023 Apr 6;13(4):e071764. doi: 10.1136/bmjopen-2023-071764 (PMC10083871; doi:10.1136/bmjopen-2023-071764)
Supplement: Author's manuscript [file bmjopen-2023-071764.draft_revisions.pdf]

BMJ Open is committed to open peer review. As part of this commitment we make the peer review history of every article we publish publicly available.

When an article is published we post the peer reviewers' comments and the authors' responses online. We also post the versions of the paper that were used during peer review. These are the versions that the peer review comments apply to.

The versions of the paper that follow are the versions that were submitted during the peer review process. They are not the versions of record or the final published versions. They should not be cited or distributed as the published version of this manuscript.

BMJ Open is an open access journal and the full, final, typeset and author-corrected version of record of the manuscript is available on our site with no access controls, subscription charges or pay-per-view fees (http://bmjopen.bmj.com).

If you have any questions on BMJ Open's open peer review process please email info.bmjopen@bmj.com

BMJ Open

Improving sleep and learning in rehabilitation after stroke, part 2 (INSPIRES2): study protocol for a randomised control trial of digital cognitive behavioural therapy (dCBT) for insomnia

Journal:	*BMJ Open*
Manuscript ID	bmjopen-2023-071764
Article Type:	Protocol
Date Submitted by the Author:	10-Jan-2023

Complete List of Authors:	Weightman, Matthew; University of Oxford Wellcome Centre for Integrative Neuroimaging, Nuffield Department of Clinical Neurosciences<br>Robinson, Barbara; University of Oxford Wellcome Centre for Integrative Neuroimaging, Nuffield Department of Clinical Neurosciences<br>Fallows, Ricky; Oxford University, Patient and Public Involvement (PPI) Author<br>Henry, Alasdair ; Big Health Ltd<br>Kyle, Simon; University of Oxford, Sleep and Circadian Neuroscience Institute<br>Garratt, Emma; Oxford Health NHS Foundation Trust<br>Pick, Anton; Oxford University Hospitals NHS Foundation Trust<br>Teal, Rachel; Oxford University Hospitals NHS Foundation Trust<br>Ajina, Sara; National Hospital for Neurology and Neurosurgery, Department of Rehabilitation and Therapy Services; University College London, Wellcome Centre for Human Neuroimaging<br>Demeyere, Nele; University of Oxford, Wolfson Centre for the Prevention of Stroke and Dementia, Nuffield Department of Clinical Neurosciences; University of Oxford, Cognitive Neuropsychology Centre, Department of Experimental Psychology<br>Espie, Colin; Big Health Ltd; Oxford University, Sleep and Circadian Neuroscience Institute, Nuffield Department of Clinical Neurosciences<br>Seymour, Ben; University of Oxford, Nuffield Department of Clinical Neurosciences<br>Johansen-Berg, Heidi; University of Oxford Wellcome Centre for Integrative Neuroimaging, Nuffield Department of Clinical Neurosciences<br>Fleming, Melanie K. ; University of Oxford Wellcome Centre for Integrative Neuroimaging, Nuffield Department of Clinical Neurosciences

Keywords:	Stroke < NEUROLOGY, SLEEP MEDICINE, REHABILITATION MEDICINE

SCHOLARONE™
Manuscripts

Improving sleep and learning in rehabilitation after stroke, part 2 (INSPIRES2): study protocol for a randomised control trial of digital cognitive behavioural therapy (dCBT) for insomnia

Authors: Matthew Weightman[1], Barbara Robinson[1], Ricky Fallows[2], Alasdair L Henry[3,4], Simon D Kyle[4], Emma Garratt[5], Anton Pick[6], Rachel Teal[7], Sara Ajina[8,9], Nele Demeyere[10,11], Colin A Espie[3,4], Ben Seymour[1], Heidi Johansen-Berg[1], Melanie K Fleming[1]

Affiliations:

[1]Wellcome Centre for Integrative Neuroimaging (WIN), Nuffield Department of Clinical Neurosciences, University of Oxford, Oxford, OX3 9DU, UK

[2]Patient and Public Involvement (PPI) Author

[3]Big Health Ltd, London, United Kingdom, WC1H 9LT

[4]Sir Jules Thorn Sleep & Circadian Neuroscience Institute, Nuffield Department of Clinical Neurosciences, University of Oxford, Oxford, OX1 3QU

[5]Oxfordshire Stroke Rehabilitation Unit (OSRU), Oxford Health NHS Foundation Trust, Oxford, United Kingdom, OX14 1AG

[6]Oxford Centre for Enablement (OCE), Oxford University Hospitals NHS Foundation Trust, Oxford, United Kingdom, OX3 7HE

[7]MRC Stroke Unit, Oxford Centre for Enablement, Oxford University Hospitals NHS Foundation Trust, Oxford, United Kingdom, OX3 7HE

[8]Department of Rehabilitation and Therapy Services, National Hospital for Neurology and Neurosurgery, University College London NHS Foundation Trust, London, United Kingdom, WC1N 3BG

[9]Wellcome Centre for Human Neuroimaging, Queen Square Institute of Neurology, University College London (UCL), London, United Kingdom, WC1N 3AR

[10]Wolfson Centre for the Prevention of Stroke and Dementia, Nuffield Department of Clinical Neurosciences, University of Oxford, Oxford, OX3 9DU

[11]Cognitive Neuropsychology Centre, Department of Experimental Psychology, University of Oxford, Oxford, OX2 6GG

For peer review only

Corresponding Author:
Matthew Weightman
Wellcome Centre for Integrative Neuroimaging (WIN),
Nuffield Department of Clinical Neurosciences,
University of Oxford,
Oxford,
OX3 9DU

matthew.weightman@ndcn.ox.ac.uk

Word Count: 3820

Keywords: *Stroke; Sleep; Rehabilitation; Memory Consolidation; Cognitive Behavioural therapy*

For peer review only

For peer review only - http://bmjopen.bmj.com/site/about/guidelines.xhtml

Abstract

Introduction

Consolidation of motor skill learning, a key component of rehabilitation post-stroke, is known to be sleep dependent. However, disrupted sleep is highly prevalent after stroke and is often associated with poor motor recovery and quality of life. Previous research has shown that digital cognitive behavioural therapy (dCBT) for insomnia can be effective at improving sleep quality after stroke. Therefore, the aim of this trial is to evaluate the potential for sleep improvement using a dCBT programme, to improve rehabilitation outcomes after stroke.

Methods and Analysis

We will conduct a parallel-arm randomised controlled trial of dCBT (Sleepio) versus treatment as usual among individuals following stroke affecting the upper limb. Up to 100 participants will be randomly allocated (2:1) into either the intervention (6-8 week dCBT) or control (continued treatment as usual) group. The primary outcome of the study will be change in insomnia symptoms pre to post intervention compared to treatment as usual. Secondary outcomes include improvement in overnight motor memory consolidation and sleep measures between intervention groups, correlations between changes in sleep behaviour and overnight motor memory consolidation in the dCBT group, and changes in symptoms of depression and fatigue between the dCBT and control groups. Analysis of covariance (ANCOVA) models and correlations will be used to analyse data from the primary and secondary outcomes.

Ethics and Dissemination

The study has received approval from the National Research Ethics Service (22/EM/0080), Health Research Authority (HRA), and Health and Care Research Wales (HCRW), IRAS ID: 306291. The results of this trial will be disseminated via

For peer review only

For peer review only - http://bmjopen.bmj.com/site/about/guidelines.xhtml

presentations at scientific conferences, peer-reviewed publication, public engagement events, stakeholder organisations, and other forms of media where appropriate.

Trial Registration:

ClinicalTrials.gov: NCT05511285, registered on 22nd August 2022.

Strengths and Limitations

- Use of an evidence-based therapy to improve sleep symptoms
- Home-based intervention, which reduces burden on participants
- Unable to blind participants to the treatment group
- Motor training component of the trial is optional and therefore will likely have a reduced sample size

For peer review only

Introduction

Stroke is one of the leading causes of long-term disability worldwide, often resulting in prolonged cognitive, motor, and emotional problems (1). Many stroke survivors also experience long-term sleep disruption, with the prevalence of post-stroke insomnia, a sleep disorder characterised by difficulties initiating and maintaining sleep, estimated to be as high as 60% (2-4). Poor sleep after stroke is frequently associated with motor/cognitive impairments, mood disorders such as anxiety and depression, and a reduced quality of life (5-7).

Motor learning – the process of learning new, or relearning old, motor skills – is an integral part of post-stroke rehabilitation (8). Improvements in motor performance not only occur during practise itself (online), but also offline during periods of rest, including sleep (9). Offline improvements, better known as consolidation, are sleep-dependent (10-14). Mechanistically, the consolidation of motor learning during sleep is thought to occur via 'reactivations' of neural activity elicited during initial learning, with stronger reactivation correlating to a greater degree of memory consolidation (15, 16). It therefore stands to reason that consolidation of motor memories after or between therapy sessions may be impaired post-stroke, via direct disruption of these biological processes that occur during sleep, thus hindering recovery. Indeed, recent results from our lab have demonstrated that sleep disturbances during rehabilitation after acquired brain injury are associated with poorer motor recovery outcomes (5). Together these findings suggest that improving sleep quality post-stroke could potentially also help to enhance rehabilitation outcomes. However, research and interventions focussed on improving symptoms of post-stroke insomnia are lacking.

Cognitive behavioural therapy (CBT) is a National Institute for Health and Care Excellence (NICE) recommended, first-line treatment for those with insomnia

For peer review only - http://bmjopen.bmj.com/site/about/guidelines.xhtml

disorder (17-19), and is supported by a wealth of evidence for improving symptoms in otherwise healthy individuals (20, 21). Whereas few patients receive access to therapist-delivered CBT due to systemic barriers (22, 23), fully-automated digital CBT (dCBT) has the potential to provide access to evidence-based treatment at scale. We, and others, have shown that the delivery of dCBT can improve insomnia symptoms (24, 25), psychological well-being (26, 27), cognitive function (28), and is also feasible to use after stroke (29). Furthermore, preliminary data suggests that dCBT is also efficacious at improving self-reported measures of sleep and mood in adult, community dwelling, stroke survivors when compared to provision of sleep hygiene information (Improving Sleep in Rehabilitation After Stroke (INSPIRES) - NCT04272892, *manuscript in preparation;* 30).

Study Objectives

Given the possible benefit of improved sleep on recovery of motoric function after stroke, the overall aim of the present study is to explore the potential for sleep improvement, via dCBT, to impact on rehabilitation outcomes post-stroke. Termed INSPIRES-2 (improving sleep and learning in rehabilitation after stroke, part 2), we will more specifically aim to:

1. Test whether symptoms of insomnia are improved in stroke survivors following dCBT in comparison to treatment as usual.
2. Assess whether overnight consolidation of motor learning is improved after the delivery of dCBT in comparison to treatment as usual.
3. Determine whether actigraphy-derived sleep measures are improved after the delivery of dCBT in comparison to treatment as usual.

For peer review only - http://bmjopen.bmj.com/site/about/guidelines.xhtml

4. Identify if changes in sleep following dCBT correlate with changes in overnight consolidation of motor learning.
5. Test for changes in symptoms of depression and fatigue after dCBT in comparison with treatment as usual.
6. Explore the feasibility and potential effect size estimates of at-home motor training following dCBT or usual care.

Methods and Analysis

Participant Recruitment

Up to 100 participants with stroke affecting the upper limb will be recruited. Participants will be identified via: National Health Service (NHS) patient identification centres, from prior study involvement at the Wellcome Centre for integrative Neuroimaging (WIN) or Cognitive Neuropsychology Centre (University of Oxford), social media, stroke support groups, and word of mouth. Participants may be identified at any time following their stroke but will not be enrolled in the study until they have been discharged from inpatient care.

Inclusion/Exclusion Criteria

Participants must be 18 years of age or above, discharged from inpatient care, and have a clinical diagnosis of stroke affecting the upper limb. Participants should, however, have sufficient movement to be able to perform the prescribed motor learning task. Individuals must be willing and able to give informed consent for participation in the study. Participants should also be interested in accessing a programme intentionally aimed at improving sleep quality and given the nature of the study, have reliable access to the internet. Individuals may not enter the study if any

For peer review only

of the following apply: diagnosis of other neurological condition affecting movement (e.g., Parkinson's Disease) or an untreated diagnosed sleep disorder (e.g., sleep apnoea); experience uncontrolled seizures; have engaged in psychological therapy for insomnia in the past 12 months; are pregnant; or have planned inpatient admission (e.g., for rehabilitation) in the next 4 months that would impact on the ability to engage with the dCBT programme.

Sample Size Distribution

Based on prior work in our lab comparing dCBT with sleep hygiene information (*manuscript in preparation*) and other studies involving dCBT in people who suffer from insomnia, we estimate a between group effect size of d = 0.7. In order to achieve this, a sample size of n = 76 will be required (α = 0.05, power = 80%, 2:1 random allocation ration dCBT:Control, two-tailed independent samples t-test). A sample size of n = 100 was selected to accommodate an approximate 25% dropout rate (determined from previous research experience). As the at-home motor training component of the trial is optional, we expect the resultant sample size to be reduced.

(Figure 1)

Trial Design

This study will be conducted as a randomised controlled trial with two parallel study arms: (1) digital cognitive behavioural therapy for insomnia (dCBT; experimental condition), and (2) treatment as usual (Treatment as usual; control condition), see Figure 1. The trial will therefore be dCBT + Treatment as usual vs Treatment as usual only and is reported in accordance with the Standard Protocol Items:

For peer review only

Recommendations for Interventional Trials checklist (31) (see online supplementary file 1). Participants will receive a version of the participant information sheet and can ask the researchers any questions they may have. Electronic informed consent is obtained from the participant (using Jisc) before any study-specific procedures are performed (online supplementary file 2).

Baseline measures of sleep will be obtained via completion of the Sleep Condition Indicator (SCI: 32) and 7-night actigraphy recordings (measuring estimated total sleep time, wakefulness after sleep onset (WASO), and sleep fragmentation index). For the actigraphy, a waterproof monitor (MotionWatch 8, CamNTech) will be delivered to participants to be worn on their least affected wrist. Participants will also complete a sleep diary (paper or digital), detailing approximately what time they attempted sleep and woke up for each of the 7 nights. Mood and fatigue will be assessed using the Patient Health Questionnaire (PHQ-9: 33) and the Fatigue Severity Scale (FSS: 34, 35), respectively.

We will assess consolidation of motor learning using the commercial rehabilitation device; Gripable (Gripable Ltd). At home, participants will complete a learning task called Balloon Buddies™ (36) at specific times of day. The task will require participants to squeeze and release the Gripable device (a hand grip dynamometer) with their affected hand in order to direct an avatar, which appears on an associated tablet, towards targets set along a predetermined path. In each attempt of the task, participants can score up to 50 points. The score participants achieve will be displayed on the screen at the end of each task attempt. We will randomly assign participants to complete one of three different 'levels' of the task, which only differ in regard to the target path but not in difficulty. Before starting the task, the Gripable will be calibrated to each participants' individual grip and release strength/ability.

For peer review only - http://bmjopen.bmj.com/site/about/guidelines.xhtml

Participants will be instructed to first complete the task in the evening (between 8-10pm or at least 30 minutes prior to sleep). They will complete the task 10 times (recording how many targets they hit each time), lasting no longer than 30 mins. Pilot data in a previous sample of stroke survivors with upper limb impairment, indicates significant improvements in accuracy over 10 repetitions (Figure 2). They will then be asked to perform a retest the next morning (between 8-10am or at least 30 minutes after wake), completing the same task a further 5 times. Again, we will ask participants to record their scores and the retest should take less than 15 mins.

Age, sex, ethnicity, date of stroke/s, stroke symptom laterality, modified Rankin Scale (mRS), and current medications will also be collected. Additionally, we will use the STOP-Bang questionnaire (37) to assess the potential risk of obstructive sleep apnoea (OSA) and a further sleep disorder questionnaire (38) to screen for sleep conditions other than insomnia. All demographic information will be collected and stored using online questionnaires (Jisc) or over the phone depending on the participants' preference. Additionally, all equipment will either be sent to the participants' homes or delivered by a researcher (depending on location and preference) and will be collected in person upon completion or return postage will be organised.

(Figure 2)

Randomisation and Blinding

Following baseline assessments, participants will be randomised at a 2:1 ratio (experimental:control) using an online randomisation software (rando.la), with minimisation of between group differences in age, sex, baseline SCI score and time

For peer review only

since stroke (months). Once randomised, each participant will be assigned a sequentially numbered study ID. It is not possible to blind the researchers involved in the day-to-day running of the study or the participants to their intervention group. Analysis will be carried out by an individual not involved in day-to-day research activity and not explicitly informed of the experimental condition.

Intervention

For the intervention group, participants will be asked to continue any of their usual treatments. In addition to this, they will be provided with access to the commercially available dCBT programme, Sleepio (Big Health Ltd). The programme involves 6 sessions of automated dCBT, with a virtual professor, and access to a daily online sleep diary. dCBT sessions become available 7 days following completion of the previous session. Participants may also interact with an online community and library of resources. Participants will be provided with a web link to the programme and will need to sign up by entering their name, email address, and a predetermined participant ID. As part of the dCBT programme, participants will be required to complete a daily online sleep diary for the duration of the programme and watch weekly videos pertaining to dCBT. Based on feedback from previous studies, they will also receive a booklet with additional details that may be useful for accessing the programme.

The control group will be asked to continue to adhere to their treatment as usual, with no further intervention measures specific to the research. The exact nature of this treatment will vary across participants. At post-intervention we will ask whether participants in the control group received any treatment for their sleep. Participants in

For peer review only

the control group will be provided the opportunity to access the dCBT programme after completing the study, however, no data will be recorded from this.

Post-Intervention

Approximately 10 weeks following randomisation, assessments of sleep (SCI, 7-night actigraphy, sleep diary), mood (PHQ-9), fatigue (FSS), and motor consolidation (Gripable task) will be repeated. Participants will be randomly assigned a different level of motor learning task so that the movement pattern is different to that which they learnt at baseline. Any participants who have not completed the dCBT programme by 10 weeks (or do not wish to complete it) will still be given the opportunity to complete the post-intervention assessments. Once completed, any equipment will be picked up by a researcher or return postage organised (dependent on participant preference, location and desire to complete voluntary training, see next section).

Optional Motor Training

Before completion of the post-intervention assessments, all participants – regardless of group allocation – will be offered 4 weeks of home-based upper limb training. This training will involve daily use of the Gripable rehabilitation device and target a wide range of hand and arm movements. Participants will be asked to attempt to perform 1 hour of training per day, either in one session or broken up into shorter sessions throughout the day. Depending on their level of impairment, participants will be instructed by a researcher which games on the device will be most suitable and how to access them, but participants are free to explore the device as they wish. We will also ask participants to complete a training diary to record how many times they use

For peer review only - http://bmjopen.bmj.com/site/about/guidelines.xhtml

the device each day and to estimate how long they spent practicing each day in total. Participants access the device through a research account, which allows the research team access to a dashboard of data. We will utilise the dashboard information to record the amount of time spent on each task and the number of movement repetitions performed each day. If participants decide to engage in this training, assessments of upper limb function will be conducted using the Action Research Arm Test (ARAT: 39), Box and Blocks Test (BBT: 40), The Nine-Hole Peg Test (9HPT: 41), and range of movement using the Gripable device (pronation/supination, wrist flexion/extension, radial/ulnar deviation and grip squeeze (strength) and release (%)). These assessments will be collected in-person, during the post-intervention visit when actigraphy and Gripable devices are delivered and repeated approximately 4 weeks later after completion of the training programme. Data from the optional training intervention will help to inform protocols and effect-size estimates of future studies combining sleep improvement and at-home rehabilitation programmes.

Outcome Measures

The primary outcome of this study will be self-reported insomnia symptoms pre to post intervention compared to treatment as usual, measured via SCI scores (32). SCI data will be collected during baseline assessments and approximately 10 weeks after randomisation, following either the dCBT intervention or treatment as usual. SCI has been shown to be a robust screening tool for the clinical evaluation of insomnia, with an estimated magnitude change of 7 scale points representing a meaningful improvement in insomnia symptoms (42). Higher values on the SCI

For peer review only - http://bmjopen.bmj.com/site/about/guidelines.xhtml

indicate fewer symptoms of insomnia and a score of 16 or below suggests probable insomnia (32).

Secondary outcome measures include: change in overnight motor memory consolidation between groups; changes in actigraphy-derived sleep measures (estimated total sleep time, WASO, and sleep fragmentation index) between groups; correlations between changes in SCI and actigraphy-derived sleep measures and overnight motor memory consolidation in the dCBT group; and changes in symptoms of depression and fatigue between the dCBT and control groups. Overnight motor consolidation will be determined by changes in performance on the Gripable motor learning task from evening training to morning retest (43), measured at baseline and approximately 10 weeks following randomisation. Actigraphy-based sleep measures at baseline and approximately 10 weeks following randomisation will be extracted and analysed using custom software, MotionWare (CamNTech Ltd) and corroborated with sleep diary entries. The PHQ-9 and FSS, measured at baseline and approximately 10 weeks following randomisation, will be used to measure changes in depression and fatigue between the dCBT and control groups. In addition to these secondary outcomes, we intend to measure the adherence and efficacy of at-home motor training during the optional intervention as an exploratory outcome. Training diaries and changes in upper limb function (ARAT, BBT and 9HPT scores) will help to determine feasibility and potential effect size estimates.

Data Analysis

Primary Outcome

An analysis of covariance (ANCOVA) will be used to test whether self-reported symptoms of insomnia are improved for the dCBT group in comparison to the control

For peer review only

group. The model will test for differences in SCI scores between groups at the post-intervention time point (~10 weeks following randomisation), with baseline score added as a covariate. All ANCOVAs will be run in general linear model format.

Secondary/Exploratory Outcomes

To test for changes in motor consolidation, we will determine the difference in performance from the end of evening training relative to the morning retest on the Gripable motor learning task at each time point. To determine if motor consolidation improves for the dCBT group compared to control, we will use an ANCOVA. The model will test for between group differences at post-intervention (~10 weeks following randomisation), with baseline consolidation score as a covariate. Changes in actigraphy-derived sleep measures will also be analysed using an ANCOVA, testing for differences between groups at 10 weeks following randomisation with baseline scores added as covariates. To explore whether the magnitude of changes in sleep measures relate to changes in motor consolidation, we will use correlational analysis (either Spearman's or Pearson's correlations as appropriate), testing for associations between changes in SCI and actigraphy-derived sleep measures (estimated total sleep time, WASO, and sleep fragmentation index) with changes in motor consolidation. We will include covariates such as age, sex, mood etc. as required. Changes in symptoms of depression and fatigue in the dCBT group compared to the control group will also be analysed using an ANCOVA to examine differences between groups on the PHQ-9 and FSS at follow up, with baseline score added as a covariate.

Patient and Public Involvement (PPI)

For peer review only

A PPI contributor (RF) has been involved in the setup of the study, including providing feedback on the dCBT programme and assisting in interviewing/appointing the research assistant who will be responsible for day-to-day management of the study. They have critically reviewed the protocol and will be included as a co-author in all published material from this study.

Throughout our previous studies of sleep after stroke and brain injury, we have discussed sleep concerns with patients in hospital and in the community (e.g., at stroke user group meetings and stroke awareness day events). We have encouraged patients to try-out and provide feedback on the motor learning task at various public/patient engagement events before and throughout the design of the study. We utilised this feedback to design the instructions we give to participants and to decide on which level of difficulty would be appropriate. Stroke survivors have also been involved in reviewing participant facing documents but will not be directly involved in recruitment of study participants or conduct of the study. We will seek further assistance from PPI contributors to help with preparation of lay summaries and dissemination of our results to ensure that these are meaningfully communicated to the stroke community.

For peer review only

Study Monitoring

Regular monitoring of participants will be performed by the research team as appropriate. Data will be evaluated for compliance with the protocol and accuracy in relation to source documents. The study may also be monitored or audited by responsible individuals from the University Sponsor and the NHS Trust(s).

At 10 weeks following randomisation we will check with participants for the potential occurrence of any adverse effects. Any adverse events that occur during

For peer review only - http://bmjopen.bmj.com/site/about/guidelines.xhtml

enrolment in the trial will be documented and participants will be advised on the best course of action. This may include withdrawing from certain assessments or contacting their General Practitioner (GP). Expected minor adverse effects (AEs) include: mild skin irritation due to wearing the actigraphy monitor, tiredness/worsening of sleep as part of the sleep restriction included in the dCBT programme, minor distress caused by completing sleep and mood questionnaires etc.

There are no anticipated serious adverse events (SAEs) associated with this trial. Any serious adverse events (SAE) that occur will be reported to the appropriate research ethics committee (REC), where in the opinion of the Chief Investigator the event was 'related' (resulted from administration of any of the research procedures) and 'unexpected' in relation to those procedures. Reports of related and unexpected SAEs will be submitted within 15 working days of the Chief Investigator becoming aware of the event, using the Health Research Authority (HRA) report of serious adverse event form (see HRA website).

Data Management

Access will be granted to authorised representatives from the Sponsor and host institution for monitoring and/or audit of the study. The participant will be referred to by their study ID, not by name, on all documents apart from the consent form. All trial data will be entered on paper and/or an electronic document (e.g., Microsoft Excel) and stored safely in confidential conditions.

Early Discontinuation/Withdrawal

For peer review only

During the trial a participant may choose to withdraw early at any time. In addition, an investigator may discontinue a participant from the trial if considered necessary for any reason including, but not limited to: ineligibility (either arising during the study or retrospectively having been overlooked at screening), significant protocol deviation*, s*ignificant non-compliance with study requirements, medical instability, and subsequent stroke affecting motor function or requiring readmission. The type of withdrawal and reason for withdrawal will be recorded. If permitted and appropriate, data obtained up until the point of withdrawal to be retained for use in the study analysis. No further data or samples would be collected after withdrawal. Withdrawn participants will be replaced providing there is sufficient time to conduct all follow up assessments within the study period.

Ethics and Dissemination

This study has received both Health Research Authority (HRA) and Health and Care Research Wales (HCRW) approval (IRAS ID: 306291). The study has also received approval from the National Research Ethics Service (22/EM/0080). The trial has been registered on a publicly accessible database prior to the recruitment of the first participant (clinicaltrials.gov NCT05511285). Results will be uploaded to clinicaltrials.gov within 12 months of the end of trial declaration by the CI or their delegate and disseminated via presentations at scientific conferences, peer-reviewed publication, public engagement events, stakeholder organisations, and other forms of media where appropriate. The investigators will be involved in reviewing drafts of the manuscripts, abstracts, press releases, and any other publications arising from the study.

For peer review only - http://bmjopen.bmj.com/site/about/guidelines.xhtml

Discussion

The ultimate aim of the present trial is to investigate the potential for improved sleep via the delivery of a digital CBT for insomnia programme, Sleepio, and examine its impact on motor learning and recovery after stroke. It is hoped that results of this trial will add to our understanding of the role of sleep in motor memory consolidation after stroke and inform future rehabilitation strategies.

In recent years, a large body of evidence has emerged supporting the crucial role of offline processes, especially those associated with sleep, for memory consolidation (14, 16, 44, 45). There is also appreciable evidence suggesting that sleep is disrupted after stroke (46-48), with those who have poorer sleep show worse rehabilitation outcomes (5, 49). Whether this relationship exists because impaired sleep post-stroke directly disrupts memory consolidation processes or reflects other factors that influence sleep and clinical outcomes e.g., depression, is yet unknown. Therefore, this study will not only help to determine the efficacy of a dCBT programme for improving sleep in stroke patients, in comparison to usual care, but also reveal if any changes in sleep measures are associated with enhanced motor memory consolidation. These results will hopefully pave the way for further study of which, if any, sleep-dependent processes of memory consolidation may be identified and targeted to improve rehabilitation outcomes, and highlight/improve clinical guidelines regarding sleep therapy after brain injury.

For peer review only

Acknowledgements

We would like to thank the NIHR CRN Stroke for their support in setting up, recruiting for, and facilitating the trial.

Author Contributions

MKF conceived and designed the protocol with input from MW, BR, RF, CAE, SDK, ALH, BS, and HJB. EG, AP, RT, SA, and ND facilitate recruitment and data collection. MW and BR are involved in the day-to-day running of the trial. MW drafted the manuscript. All authors edited, revised and approved the final version of the manuscript.

Funding

This study is funded by Guarantors of Brain (Fellowship to MKF) and the Wellcome Trust (Principal Research Fellowship to HJB; 222446/Z/21/Z) and supported by the NIHR Oxford (Oxford University Hospitals) and Oxford Health Biomedical Research Centres (BRC). These funding sources had no role in the design of this study and will not have any role during its execution, analyses, or interpretation of data.

Competing Interests

CAE is co-founder and Chief Scientist of Big Health Ltd., is salaried and is a shareholder. ALH is employed by Big Health Ltd, is salaried and a shareholder. Big Health Ltd. are supplying Sleepio free of charge to the study.

Rights Retention

For peer review only

For peer review only - http://bmjopen.bmj.com/site/about/guidelines.xhtml

This research is funded in whole, or in part, by the Wellcome Trust [Grant number 222446/Z/21/Z]. For the purpose of open access, the author has applied a CCBY public copyright license to any Author Accepted Manuscript version arising from this submission.

For peer review only

References

(1) Feigin VL, Brainin M, Norrving B, Martins S, Sacco RL, Hacke W, et al. World Stroke Organization (WSO): global stroke fact sheet 2022. *International Journal of Stroke*. 2022;17(1):18-29. https://doi.org/10.1177/17474930211065917

(2) Gottlieb E, Landau E, Baxter H, Werden E, Howard ME, Brodtmann A. The bidirectional impact of sleep and circadian rhythm dysfunction in human ischaemic stroke: a systematic review. *Sleep Medicine Reviews*. 2019;45:54-69. doi: https://doi.org/10.1016/j.smrv.2019.03.003

(3) Baylan S, Griffiths S, Grant N, Broomfield NM, Evans JJ, Gardani M. Incidence and prevalence of post-stroke insomnia: a systematic review and meta-analysis. *Sleep medicine reviews*. 2020;49:101222. https://doi.org/10.1016/j.smrv.2019.101222

(4) Hasan F, Gordon C, Wu D, Huang HC, Yuliana LT, Susatia B, et al. Dynamic prevalence of sleep disorders following stroke or transient ischemic attack: systematic review and meta-analysis. *Stroke*. 2021;52(2):655-63. https://doi.org/10.1161/STROKEAHA.120.029847

(5) Fleming MK, Smejka T, Henderson Slater D, van Gils V, Garratt E, Yilmaz Kara E, et al. Sleep disruption after brain injury is associated with worse motor outcomes and slower functional recovery. *Neurorehabilitation and Neural Repair*. 2020;34(7):661- 71. https://doi.org/10.1177/154596832092966

(6) Fulk GD, Boyne P, Hauger M, Ghosh R, Romano S, Thomas J, et al. The impact of sleep disorders on functional recovery and participation following stroke: A systematic review and meta-analysis. *Neurorehabilitation and Neural Repair*. 2020;34(11):1050-61. doi: https://doi.org/10.1177/1545968320962501

(7) Fleming MK, Smejka T, Henderson Slater D, Chiu EG, Demeyere N, Johansen-Berg H. Self-reported and objective sleep measures in stroke survivors with incomplete motor recovery at the chronic stage. *Neurorehabilitation and Neural Repair*. 2021;35(10):851-60. https://doi.org/10.1177/15459683211029889

(8) Krakauer JW. Motor learning: its relevance to stroke recovery and neurorehabilitation. *Current opinion in neurology*. 2006;19(1):84-90. doi: 10.1097/01.wco.0000200544.29915.cc

(9) Walker MP, Brakefield T, Seidman J, Morgan A, Hobson JA, Stickgold R. Sleep and the time course of motor skill learning. *Learning & memory*. 2003;10(4):275-84. doi: 10.1101/lm.58503

For peer review only

For peer review only - http://bmjopen.bmj.com/site/about/guidelines.xhtml

(10) Stickgold R, James L, Hobson JA. Visual discrimination learning requires sleep after training. *Nature neuroscience*. 2000;3(12):1237-8. doi: https://doi.org/10.1038/81756

(11) Stickgold R, Whidbee D, Schirmer B, Patel V, Hobson JA. Visual discrimination task improvement: A multi-step process occurring during sleep. *Journal of cognitive neuroscience*. 2000;12(2):246-54. https://doi.org/10.1162/089892900562075

(12) Fischer S, Hallschmid M, Elsner AL, Born J. Sleep forms memory for finger skills. *Proceedings of the National Academy of Sciences*. 2002;99(18):11987-91. doi: https://doi.org/10.1073/pnas.182178199

(13) Huber R, Felice Ghilardi M, Massimini M, Tononi G. Local sleep and learning. *Nature*. 2004;430(6995):78-81. doi: https://doi.org/10.1038/nature02663

(14) GudbergC,WulffK,Johansen-BergH.Sleep-dependentmotormemoryconsolidation in older adults depends on task demands. *Neurobiology of aging*. 2015;36(3):1409-16. doi: https://doi.org/10.1016/j.neurobiolaging.2014.12.014

(15) Deuker L, Olligs J, Fell J, Kranz TA, Mormann F, Montag C, et al. Memory consolidation by replay of stimulus-specific neural activity. *Journal of Neuroscience*. 2013;33(49):19373-83. https://doi.org/10.1523/JNEUROSCI.0414-13.2013

(16) Ramanathan DS, Gulati T, Ganguly K. Sleep-dependent reactivation of ensembles in motor cortex promotes skill consolidation. *PLoS biology*. 2015;13(9):e1002263. doi: https://doi.org/10.1371/journal.pbio.1002263

(17) Qaseem A, Kansagara D, Forciea MA, Cooke M, Denberg TD, of the American College of Physicians* CGC. Management of chronic insomnia disorder in adults: a clinical practice guideline from the American College of Physicians. *Annals of internal medicine*. 2016;165(2):125-33. https://doi.org/10.7326/M15-2175

(18) Riemann D, Baglioni C, Bassetti C, Bjorvatn B, Dolenc Groselj L, Ellis JG, et al. European guideline for the diagnosis and treatment of insomnia. *Journal of sleep research*. 2017;26(6):675-700. https://doi.org/10.1111/jsr.12594

(19) Edinger JD, Arnedt JT, Bertisch SM, Carney CE, Harrington JJ, Lichstein KL, et al. Behavioral and psychological treatments for chronic insomnia disorder in adults: an American Academy of Sleep Medicine clinical practice guideline. *Journal of Clinical Sleep Medicine*. 2021;17(2):255-62. https://doi.org/10.5664/jcsm.8986

(20) Trauer JM, Qian MY, Doyle JS, Rajaratnam SM, Cunnington D. Cognitive behavioral therapy for chronic insomnia: a systematic review and meta-analysis. *Annals of internal medicine*. 2015;163(3):191-204. doi: https://doi.org/10.7326/M14-2841

For peer review only

For peer review only - http://bmjopen.bmj.com/site/about/guidelines.xhtml

(21) Van Straten A, van der Zweerde T, Kleiboer A, Cuijpers P, Morin CM, Lancee J. Cognitive and behavioral therapies in the treatment of insomnia: a meta-analysis. *Sleep medicine reviews*. 2018;38:3-16. doi: https://doi.org/10.1016/j.smrv.2017.02.001

(22) Koffel E, Bramoweth AD, Ulmer CS. Increasing access to and utilization of cognitive behavioral therapy for insomnia (CBT-I): a narrative review. *Journal of General Internal Medicine*. 2018;33(6):955-62. https://doi.org/10.1007/s11606-018-4390-1

(23) Thomas A, Grandner M, Nowakowski S, Nesom G, Corbitt C, Perlis ML. Where are the behavioral sleep medicine providers and where are they needed? A geographic assessment. *Behavioral sleep medicine*. 2016;14(6):687-98. https://doi.org/10.1080/15402002.2016.1173551

(24) Espie CA, Kyle SD, Williams C, Ong JC, Douglas NJ, Hames P, et al. A randomized, placebo-controlled trial of online cognitive behavioral therapy for chronic insomnia disorder delivered via an automated media-rich web application. *Sleep*. 2012;35(6):769-81. doi: https://doi.org/10.5665/sleep.1872

(25) Luik AI, Kyle SD, Espie CA. Digital cognitive behavioral therapy (dCBT) for insomnia: a state-of-the-science review. *Current Sleep Medicine Reports*. 2017;3(2):48-56. https://doi.org/10.1007/s40675-017-0065-4

(26) Espie CA, Emsley R, Kyle SD, Gordon C, Drake CL, Siriwardena AN, et al. Effect of digital cognitive behavioral therapy for insomnia on health, psychological well- being, and sleep-related quality of life: a randomized clinical trial. *JAMA psychiatry*. 2019;76(1):21-30. doi: 10.1001/jamapsychiatry.2018.2745

(27) Henry AL, Miller CB, Emsley R, Sheaves B, Freeman D, Luik AI, et al. Insomnia as a mediating therapeutic target for depressive symptoms: a sub-analysis of participant data from two large randomized controlled trials of a digital sleep intervention. *Journal of sleep research*. 2021;30(1):e13140. https://doi.org/10.1111/jsr.13140

(28) Kyle SD, Hurry ME, Emsley R, Marsden A, Omlin X, Juss A, et al. The effects of digital cognitive behavioral therapy for insomnia on cognitive function: a randomized controlled trial. *Sleep*. 2020;43(9):zsaa034. doi: https://doi.org/10.1093/sleep/zsaa034

(29) Smejka T, Henry AL, Wheatley C, Espie CA, Johansen-Berg H, Fleming MK. A qualitative examination of the usability of a digital cognitive behavioral therapy for insomnia program after stroke. *Brain Injury*. 2022:1-8. doi: https://doi.org/10.1080/02699052.2022.2034182

For peer review only

(30) Fleming MK, Smejka T, Macey E, Henry AL, Luengo-Fernandez R, Espie CA, Johansen-Berg H. Improving sleep in rehabilitation after stroke (INSPIRES). ESOC 2022 Abstract Book. *European Stroke Journal*. 2022;7(1_suppl):3-545. doi:10.1177/23969873221087559

(31) Chan AW, Tetzlaff JM, Altman DG, Laupacis A, Gøtzsche PC, Krleža-Jeri´c K, et al. SPIRIT 2013 statement: defining standard protocol items for clinical trials. *Annals of internal medicine*. 2013;158(3):200-7. https://doi.org/10.7326/0003-4819-158-3-201302050-00583

(32) Espie CA, Kyle SD, Hames P, Gardani M, Fleming L, Cape J. The Sleep Condition Indicator: a clinical screening tool to evaluate insomnia disorder. *BMJ open*. 2014;4(3):e004183. doi: doi:10.1136/bmjopen-2013-004183

(33) Kroenke K, Spitzer RL, Williams JB. The PHQ-9: validity of a brief depression severity measure. *Journal of general internal medicine*. 2001;16(9):606-13. https://doi.org/10.1046/j.1525-1497.2001.016009606.x

(34) Schwartz JE, Jandorf L, Krupp LB. The measurement of fatigue: a new instrument. *Journal of psychosomatic research*. 1993;37(7):753-62. https://doi.org/10.1016/0022-3999(93)90104-N

(35) Lerdal A, Kottorp A. Psychometric properties of the Fatigue Severity Scale—Rasch analyses of individual responses in a Norwegian stroke cohort. *International journal of nursing studies*. 2011;48(10):1258-65. https://doi.org/10.1016/j.ijnurstu.2011.02.019

(36) Mace M, Kinany N, Rinne P, Rayner A, Bentley P, Burdet E. Balancing the playing field: collaborative gaming for physical training. *Journal of neuroengineering and rehabilitation*. 2017;14(1):1-18. https://doi.org/10.1186/s12984-017-0319-x

(37) Chung F, Abdullah HR, Liao P. STOP-Bang questionnaire: a practical approach to screen for obstructive sleep apnea. *Chest*. 2016;149(3):631-8. https://doi.org/10.1378/chest.15-0903

(38) Wilson SJ, Nutt D, Alford C, Argyropoulos S, Baldwin DS, Bateson A, et al. British Association for Psychopharmacology consensus statement on evidence-based treatment of insomnia, parasomnias and circadian rhythm disorders. *Journal of psychopharmacology*. 2010;24(11):1577-601. https://doi.org/10.1177/0269881110379307

For peer review only - http://bmjopen.bmj.com/site/about/guidelines.xhtml

(39) Lyle RC. A performance test for assessment of upper limb function in physical rehabilitation treatment and research. *International journal of rehabilitation research*. 1981;4(4):483-92.

(40) Mathiowetz V, Volland G, Kashman N, Weber K. Adult norms for the Box and Block Test of manual dexterity. *The American journal of occupational therapy*. 1985;39(6):386-91. doi: https://doi.org/10.5014/ajot.39.6.386

(41) Mathiowetz V, Weber K, Kashman N, Volland G. Adult norms for the nine hole peg test of finger dexterity. *The Occupational Therapy Journal of Research*. 1985;5(1):24- 38. doi: https://doi.org/10.1177/153944928500500102

(42) Espie CA, Farias Machado P, Carl JR, Kyle SD, Cape J, Siriwardena AN, et al. The Sleep Condition Indicator: reference values derived from a sample of 200 000 adults. *Journal of sleep research*. 2018;27(3):e12643. https://doi.org/10.1111/jsr.12643

(43) Robertson EM, Pascual-Leone A, Miall RC. Current concepts in procedural consolidation. *Nature Reviews Neuroscience*. 2004;5(7):576-82. https://doi.org/10.1038/nrn1426

(44) Walker MP, Brakefield T, Morgan A, Hobson JA, Stickgold R. Practice with sleep makes perfect: sleep-dependent motor skill learning. *Neuron*. 2002;35(1):205-11. doi: https://doi.org/10.1016/S0896-6273(02)00746-8

(45) Belal S, Cousins J, El-Deredy W, Parkes L, Schneider J, Tsujimura H, et al. Identification of memory reactivation during sleep by EEG classification. *Neuroimage*. 2018;176:203-14. https://doi.org/10.1016/j.neuroimage.2018.04.029

(46) Duss SB, Seiler A, Schmidt MH, Pace M, Adamantidis A, Müri RM, et al. The role of sleep in recovery following ischemic stroke: a review of human and animal data. *Neurobiology of sleep and circadian rhythms*. 2017;2:94-105. https://doi.org/10.1016/j.nbscr.2016.11.003

(47) Sterr A, Kuhn M, Nissen C, Ettine D, Funk S, Feige B, et al. Post-stroke insomnia in community-dwelling patients with chronic motor stroke: Physiological evidence and implications for stroke care. *Scientific Reports*. 2018;8(1):1-9. doi: https://doi.org/10.1038/s41598-018-26630-y

(48) Khot SP, Morgenstern LB. Sleep and stroke. *Stroke*. 2019;50(6):1612-7. doi: https://doi.org/10.1161/STROKEAHA.118.023553

(49) Iddagoda MT, Inderjeeth CA, Chan K, Raymond WD. Post-stroke sleep disturbances and rehabilitation outcomes: a prospective cohort study. *Internal Medicine Journal*. 2020;50(2):208-13. doi: https://doi.org/10.1111/imj.14372

For peer review only

665 Walker, M. P., Brakefield, T., Seidman, J., Morgan, A., Hobson, J. A., & Stickgold, R.
666 (2003). Sleep and the time course of motor skill learning. *Learning & memory*, *10*(4),
667 275–284. doi: 10.1101/lm.58503

668 Wilson, S. J., Nutt, D. J., Alford, C., Argyropoulos, S. V., Baldwin, D. S., Bateson, A.
669 N., ... & Wade, A. G. (2010). British Association for Psychopharmacology consensus
670 statement on evidence-based treatment of insomnia, parasomnias and circadian
671 rhythm disorders. *Journal of psychopharmacology*, *24*(11), 1577-1601.
672 https://doi.org/10.1177/0269881110379307

For peer review only

For peer review only - http://bmjopen.bmj.com/site/about/guidelines.xhtml

Figure Legends

Figure 1: *Flow chart of the trial, depicting patient recruitment, screening, intervention, assessments, and optional motor training. Sleep Condition Indicator; SCI, Modified Rankin Scale; mRS, Obstructive Sleep Apnoea Questionnaire; STOP-Bang, Patient Health Questionnaire; PHQ-9, Fatigue Severity Scale; FSS, Digital Cognitive behavioural Therapy; dCBT, Action Research Arm Test; ARAT, Box and Blocks Test; BBT, Nine-Hole Peg Test; 9HPT.*

Figure 2: *Number of targets hit (maximum 50) per repetition of the Gripable motor learning task in a sample of stroke survivors with upper limb impairment. Mean scores indicated by filled circles ± standard error (sample size: n = 25).*

For peer review only

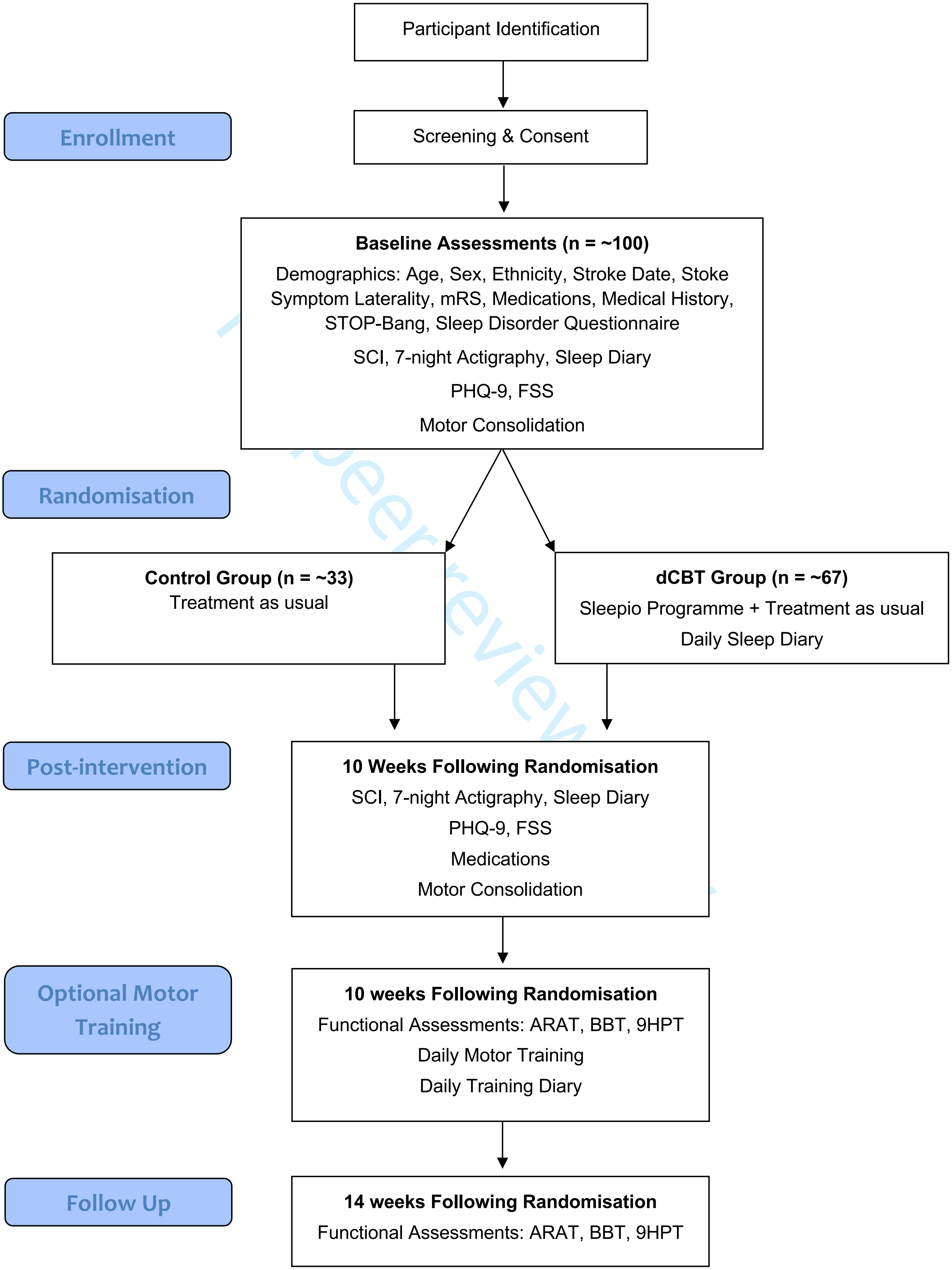
Participant Identification
Enrollment
Screening & Consent
Baseline Assessments (n = ~100)
Demographics: Age, Sex, Ethnicity, Stroke Date, Stoke Symptom Laterality, mRS, Medications, Medical History, STOP-Bang, Sleep Disorder Questionnaire
SCI, 7-night Actigraphy, Sleep Diary
PHQ-9, FSS
Motor Consolidation
Randomisation
Control Group (n = ~33)
Treatment as usual
dCBT Group (n = ~67)
Sleepio Programme + Treatment as usual
Daily Sleep Diary
Post-intervention
10 Weeks Following Randomisation
SCI, 7-night Actigraphy, Sleep Diary
PHQ-9, FSS
Medications
Motor Consolidation
Optional Motor Training
10 weeks Following Randomisation
Functional Assessments: ARAT, BBT, 9HPT
Daily Motor Training
Daily Training Diary
Follow Up
14 weeks Following Randomisation
Functional Assessments: ARAT, BBT, 9HPT

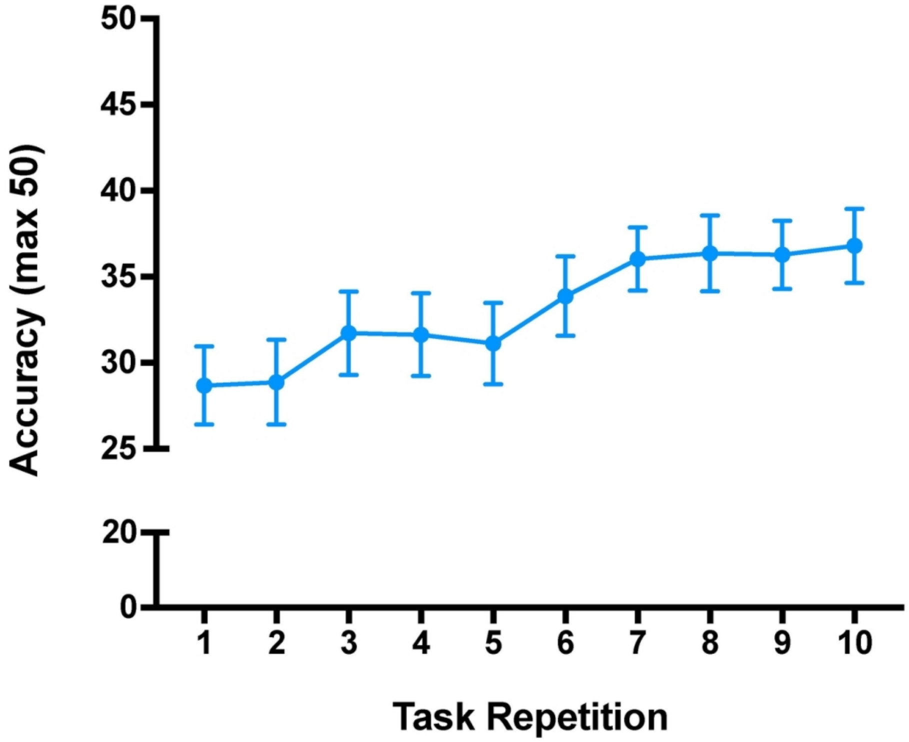

Number of targets hit (maximum 50) per repetition of the Gripable motor learning task in a sample of stroke survivors with upper limb impairment. Mean scores indicated by filled circles ± standard error (sample size: n = 25).

127x98mm (300 x 300 DPI)

Reporting checklist for protocol of a clinical trial.

Based on the SPIRIT guidelines.

Instructions to authors

Complete this checklist by entering the page numbers from your manuscript where readers will find each of the items listed below.

Your article may not currently address all the items on the checklist. Please modify your text to include the missing information. If you are certain that an item does not apply, please write "n/a" and provide a short explanation.

Upload your completed checklist as an extra file when you submit to a journal.

In your methods section, say that you used the SPIRITreporting guidelines, and cite them as:

Chan A-W, Tetzlaff JM, Gøtzsche PC, Altman DG, Mann H, Berlin J, Dickersin K, Hróbjartsson A, Schulz KF, Parulekar WR, Krleža-Jerić K, Laupacis A, Moher D. SPIRIT 2013 Explanation and Elaboration: Guidance for protocols of clinical trials. BMJ. 2013;346:e7586

		Reporting Item	Page Number
**Administrative information**			
Title	#1	Descriptive title identifying the study design, population, interventions, and, if applicable, trial acronym	1
Trial registration	#2a	Trial identifier and registry name. If not yet registered, name of intended registry	4
Trial registration: data set	#2b	All items from the World Health Organization Trial Registration Data Set	n/a
Protocol version	#3	Date and version identifier	4
Funding	#4	Sources and types of financial, material, and other support	26

For peer review only

Roles and responsibilities: contributorship	#5a	Names, affiliations, and roles of protocol contributors	1 & 26
Roles and responsibilities: sponsor contact information	#5b	Name and contact information for the trial sponsor	1
Roles and responsibilities: sponsor and funder	#5c	Role of study sponsor and funders, if any, in study design; collection, management, analysis, and interpretation of data; writing of the report; and the decision to submit the report for publication, including whether they will have ultimate authority over any of these activities	26
Roles and responsibilities: committees	#5d	Composition, roles, and responsibilities of the coordinating centre, steering committee, endpoint adjudication committee, data management team, and other individuals or groups overseeing the trial, if applicable (see Item 21a for data monitoring committee)	16-18 & 26
**Introduction**			
Background and rationale	#6a	Description of research question and justification for undertaking the trial, including summary of relevant studies (published and unpublished) examining benefits and harms for each intervention	5-6
Background and rationale: choice of comparators	#6b	Explanation for choice of comparators	7
Objectives	#7	Specific objectives or hypotheses	6-7
Trial design	#8	Description of trial design including type of trial (eg, parallel group, crossover, factorial, single group),	8-10

For peer review only

		allocation ratio, and framework (eg, superiority, equivalence, non-inferiority, exploratory)	
**Methods: Participants, interventions, and outcomes**			
Study setting	#9	Description of study settings (eg, community clinic, academic hospital) and list of countries where data will be collected. Reference to where list of study sites can be obtained	9
Eligibility criteria	#10	Inclusion and exclusion criteria for participants. If applicable, eligibility criteria for study centres and individuals who will perform the interventions (eg, surgeons, psychotherapists)	7-8
Interventions: description	#11a	Interventions for each group with sufficient detail to allow replication, including how and when they will be administered	11-12
Interventions: modifications	#11b	Criteria for discontinuing or modifying allocated interventions for a given trial participant (eg, drug dose change in response to harms, participant request, or improving / worsening disease)	17-18
Interventions: adherance	#11c	Strategies to improve adherence to intervention protocols, and any procedures for monitoring adherence (eg, drug tablet return; laboratory tests)	16-18
Interventions: concomitant care	#11d	Relevant concomitant care and interventions that are permitted or prohibited during the trial	8 & 11-12
Outcomes	#12	Primary, secondary, and other outcomes, including the specific measurement variable (eg, systolic blood pressure), analysis metric (eg, change from baseline, final value, time to event), method of aggregation (eg, median, proportion), and time point for each outcome. Explanation of the clinical relevance of chosen efficacy and harm outcomes is strongly recommended	13-14

For peer review only

Participant timeline	#13	Time schedule of enrolment, interventions (including any run-ins and washouts), assessments, and visits for participants. A schematic diagram is highly recommended (see Figure)	Figure 1
Sample size	#14	Estimated number of participants needed to achieve study objectives and how it was determined, including clinical and statistical assumptions supporting any sample size calculations	8
Recruitment	#15	Strategies for achieving adequate participant enrolment to reach target sample size	8
**Methods: Assignment of interventions (for controlled trials)**			
Allocation: sequence generation	#16a	Method of generating the allocation sequence (eg, computer-generated random numbers), and list of any factors for stratification. To reduce predictability of a random sequence, details of any planned restriction (eg, blocking) should be provided in a separate document that is unavailable to those who enrol participants or assign interventions	12
Allocation concealment mechanism	#16b	Mechanism of implementing the allocation sequence (eg, central telephone; sequentially numbered, opaque, sealed envelopes), describing any steps to conceal the sequence until interventions are assigned	10-11
Allocation: implementation	#16c	Who will generate the allocation sequence, who will enrol participants, and who will assign participants to interventions	10-11

For peer review only

Blinding (masking)	#17a	Who will be blinded after assignment to interventions (eg, trial participants, care providers, outcome assessors, data analysts), and how	10-11
Blinding (masking): emergency unblinding	#17b	If blinded, circumstances under which unblinding is permissible, and procedure for revealing a participant's allocated intervention during the trial	n/a
**Methods: Data collection, management, and analysis**			
Data collection plan	#18a	Plans for assessment and collection of outcome, baseline, and other trial data, including any related processes to promote data quality (eg, duplicate measurements, training of assessors) and a description of study instruments (eg, questionnaires, laboratory tests) along with their reliability and validity, if known. Reference to where data collection forms can be found, if not in the protocol	8-10, 12-13
Data collection plan: retention	#18b	Plans to promote participant retention and complete follow-up, including list of any outcome data to be collected for participants who discontinue or deviate from intervention protocols	16-17
Data management	#19	Plans for data entry, coding, security, and storage, including any related processes to promote data quality (eg, double data entry; range checks for data values). Reference to where details of data management procedures can be found, if not in the protocol	17
Statistics: outcomes	#20a	Statistical methods for analysing primary and secondary outcomes. Reference to where other details of the statistical analysis plan can be found, if not in the protocol	14-15

For peer review only

Statistics: additional analyses	#20b	Methods for any additional analyses (eg, subgroup and adjusted analyses)	14-15
Statistics: analysis population and missing data	#20c	Definition of analysis population relating to protocol non-adherence (eg, as randomised analysis), and any statistical methods to handle missing data (eg, multiple imputation)	n/a
**Methods: Monitoring**			
Data monitoring: formal committee	#21a	Composition of data monitoring committee (DMC); summary of its role and reporting structure; statement of whether it is independent from the sponsor and competing interests; and reference to where further details about its charter can be found, if not in the protocol. Alternatively, an explanation of why a DMC is not needed	n/a
Data monitoring: interim analysis	#21b	Description of any interim analyses and stopping guidelines, including who will have access to these interim results and make the final decision to terminate the trial	17-18
Harms	#22	Plans for collecting, assessing, reporting, and managing solicited and spontaneously reported adverse events and other unintended effects of trial interventions or trial conduct	16-17
Auditing	#23	Frequency and procedures for auditing trial conduct, if any, and whether the process will be independent from investigators and the sponsor	16-18
**Ethics and dissemination**			
Research ethics approval	#24	Plans for seeking research ethics committee / institutional review board (REC / IRB) approval	3

For peer review only

Protocol amendments	#25	Plans for communicating important protocol modifications (eg, changes to eligibility criteria, outcomes, analyses) to relevant parties (eg, investigators, REC / IRBs, trial participants, trial registries, journals, regulators)	26-27
Consent or assent	#26a	Who will obtain informed consent or assent from potential trial participants or authorised surrogates, and how (see Item 32)	8-10
Consent or assent: ancillary studies	#26b	Additional consent provisions for collection and use of participant data and biological specimens in ancillary studies, if applicable	n/a
Confidentiality	#27	How personal information about potential and enrolled participants will be collected, shared, and maintained in order to protect confidentiality before, during, and after the trial	11, 17-18
Declaration of interests	#28	Financial and other competing interests for principal investigators for the overall trial and each study site	26
Data access	#29	Statement of who will have access to the final trial dataset, and disclosure of contractual agreements that limit such access for investigators	17
Ancillary and post trial care	#30	Provisions, if any, for ancillary and post-trial care, and for compensation to those who suffer harm from trial participation	n/a
Dissemination policy: trial results	#31a	Plans for investigators and sponsor to communicate trial results to participants, healthcare professionals, the public, and other relevant groups (eg, via publication, reporting in results databases, or other data sharing arrangements), including any publication restrictions	18
Dissemination policy: authorship	#31b	Authorship eligibility guidelines and any intended use of professional writers	n/a

For peer review only

Dissemination policy: reproducible research	#31c	Plans, if any, for granting public access to the full protocol, participant-level dataset, and statistical code	26-27

Appendices

Informed consent materials	#32	Model consent form and other related documentation given to participants and authorised surrogates	Supplementary file 2
Biological specimens	#33	Plans for collection, laboratory evaluation, and storage of biological specimens for genetic or molecular analysis in the current trial and for future use in ancillary studies, if applicable	n/a

None The SPIRIT Explanation and Elaboration paper is distributed under the terms of the Creative Commons Attribution License CC-BY-NC. This checklist can be completed online using https://www.goodreports.org/, a tool made by the EQUATOR Network in collaboration with Penelope.ai

For peer review only

Dr Melanie Fleming
Sleep-win@ndcn.ox.ac.uk

Participant identification number:

CONSENT FORM

Study Title: INSPIRES-2

If you agree, please initial box

1. I confirm that I have read the information sheet dated................... (version............) for this study. I have had the opportunity to consider the information, ask questions and have had these answered satisfactorily.		
2. I understand that my participation is voluntary and that I am free to withdraw at any time without giving any reason, without my medical care or legal rights being affected.		
3. I understand that relevant sections of my medical notes and data collected during the study may be looked at by individuals from University of Oxford, from regulatory authorities and from the NHS Trust(s), where it is relevant to my taking part in this research. I give permission for these individuals to have access to my records.		
4. I agree to take part in this study.		
Additional:		
5. *I agree to be contacted about ethically approved research studies for which I may be suitable. I understand that agreeing to be contacted does not oblige me to participate in any further studies.*	Yes	No
6. *I would like the researchers to send a letter to my GP to let them know that I am taking part in this research (if yes, please give details)*	Yes	No

For peer review only - http://bmjopen.bmj.com/site/about/guidelines.xhtml

Name of Participant *Date*

When completed: 1 for participant; 1 for researcher

If you agreed for researchers to send a letter to your GP, please provide your GP details

GP name (if known):

Surgery name:

Surgery address:

For peer review only - http://bmjopen.bmj.com/site/about/guidelines.xhtml

BMJ Open

Improving sleep and learning in rehabilitation after stroke, part 2 (INSPIRES2): study protocol for a home-based randomised control trial of digital cognitive behavioural therapy (dCBT) for insomnia

Journal:	*BMJ Open*
Manuscript ID	bmjopen-2023-071764.R1
Article Type:	Protocol
Date Submitted by the Author:	17-Feb-2023
Complete List of Authors:	Weightman, Matthew; University of Oxford Wellcome Centre for Integrative Neuroimaging, Nuffield Department of Clinical Neurosciences<br>Robinson, Barbara; University of Oxford Wellcome Centre for Integrative Neuroimaging, Nuffield Department of Clinical Neurosciences<br>Fallows, Ricky; Oxford University, Patient and Public Involvement (PPI) Author<br>Henry, Alasdair ; Big Health Ltd<br>Kyle, Simon; University of Oxford, Sleep and Circadian Neuroscience Institute<br>Garratt, Emma; Oxford Health NHS Foundation Trust<br>Pick, Anton; Oxford University Hospitals NHS Foundation Trust<br>Teal, Rachel; Oxford University Hospitals NHS Foundation Trust<br>Ajina, Sara; National Hospital for Neurology and Neurosurgery, Department of Rehabilitation and Therapy Services; University College London, Wellcome Centre for Human Neuroimaging<br>Demeyere, Nele; University of Oxford, Wolfson Centre for the Prevention of Stroke and Dementia, Nuffield Department of Clinical Neurosciences; University of Oxford, Cognitive Neuropsychology Centre, Department of Experimental Psychology<br>Espie, Colin; Big Health Ltd; Oxford University, Sleep and Circadian Neuroscience Institute, Nuffield Department of Clinical Neurosciences<br>Seymour, Ben; University of Oxford, Nuffield Department of Clinical Neurosciences<br>Johansen-Berg, Heidi; University of Oxford Wellcome Centre for Integrative Neuroimaging, Nuffield Department of Clinical Neurosciences<br>Fleming, Melanie K. ; University of Oxford Wellcome Centre for Integrative Neuroimaging, Nuffield Department of Clinical Neurosciences
**Primary Subject Heading**:	Rehabilitation medicine
Secondary Subject Heading:	Neurology
Keywords:	Stroke < NEUROLOGY, SLEEP MEDICINE, REHABILITATION MEDICINE

For peer review only - http://bmjopen.bmj.com/site/about/guidelines.xhtml

Improving sleep and learning in rehabilitation after stroke, part 2 (INSPIRES2): study protocol for a home-based randomised control trial of digital cognitive behavioural therapy (dCBT) for insomnia

Authors: Matthew Weightman[1], Barbara Robinson[1], Ricky Fallows[2], Alasdair L Henry[3,4], Simon D Kyle[4], Emma Garratt[5], Anton Pick[6], Rachel Teal[7], Sara Ajina[8,9], Nele Demeyere[10,11], Colin A Espie[3,4], Ben Seymour[1], Heidi Johansen-Berg[1], Melanie K Fleming[1]

Affiliations:

[1]Wellcome Centre for Integrative Neuroimaging (WIN), Nuffield Department of Clinical Neurosciences, University of Oxford, Oxford, OX3 9DU, UK

[2]Patient and Public Involvement (PPI) Author

[3]Big Health Ltd, London, United Kingdom, WC1H 9LT

[4]Sir Jules Thorn Sleep & Circadian Neuroscience Institute, Nuffield Department of Clinical Neurosciences, University of Oxford, Oxford, OX1 3QU

[5]Oxfordshire Stroke Rehabilitation Unit (OSRU), Oxford Health NHS Foundation Trust, Oxford, United Kingdom, OX14 1AG

[6]Oxford Centre for Enablement (OCE), Oxford University Hospitals NHS Foundation Trust, Oxford, United Kingdom, OX3 7HE

[7]MRC Stroke Unit, Oxford Centre for Enablement, Oxford University Hospitals NHS Foundation Trust, Oxford, United Kingdom, OX3 7HE

[8]Department of Rehabilitation and Therapy Services, National Hospital for Neurology and Neurosurgery, University College London NHS Foundation Trust, London, United Kingdom, WC1N 3BG

[9]Wellcome Centre for Human Neuroimaging, Queen Square Institute of Neurology, University College London (UCL), London, United Kingdom, WC1N 3AR

[10]Wolfson Centre for the Prevention of Stroke and Dementia, Nuffield Department of Clinical Neurosciences, University of Oxford, Oxford, OX3 9DU

[11]Cognitive Neuropsychology Centre, Department of Experimental Psychology, University of Oxford, Oxford, OX2 6GG

For peer review only

Corresponding Author:
Matthew Weightman
Wellcome Centre for Integrative Neuroimaging (WIN),
Nuffield Department of Clinical Neurosciences,
University of Oxford,
Oxford,
OX3 9DU

matthew.weightman@ndcn.ox.ac.uk

Word Count: 3730

Keywords: *Stroke; Sleep; Rehabilitation; Memory Consolidation; Cognitive Behavioural therapy*

For peer review only

Abstract

Introduction

Consolidation of motor skill learning, a key component of rehabilitation post-stroke, is known to be sleep dependent. However, disrupted sleep is highly prevalent after stroke and is often associated with poor motor recovery and quality of life. Previous research has shown that digital cognitive behavioural therapy (dCBT) for insomnia can be effective at improving sleep quality after stroke. Therefore, the aim of this trial is to evaluate the potential for sleep improvement using a dCBT programme, to improve rehabilitation outcomes after stroke.

Methods and Analysis

We will conduct a parallel-arm randomised controlled trial of dCBT (Sleepio) versus treatment as usual among individuals following stroke affecting the upper limb. Up to 100 participants will be randomly allocated (2:1) into either the intervention (6-8 week dCBT) or control (continued treatment as usual) group. The primary outcome of the study will be change in insomnia symptoms pre to post intervention compared to treatment as usual. Secondary outcomes include improvement in overnight motor memory consolidation and sleep measures between intervention groups, correlations between changes in sleep behaviour and overnight motor memory consolidation in the dCBT group, and changes in symptoms of depression and fatigue between the dCBT and control groups. Analysis of covariance (ANCOVA) models and correlations will be used to analyse data from the primary and secondary outcomes.

Ethics and Dissemination

The study has received approval from the National Research Ethics Service (22/EM/0080), Health Research Authority (HRA), and Health and Care Research Wales (HCRW), IRAS ID: 306291. The results of this trial will be disseminated via

For peer review only - http://bmjopen.bmj.com/site/about/guidelines.xhtml

presentations at scientific conferences, peer-reviewed publication, public engagement events, stakeholder organisations, and other forms of media where appropriate.

Trial Registration:

ClinicalTrials.gov: NCT05511285, registered on 22nd August 2022.

Strengths and Limitations

- Use of an evidence-based therapy to improve sleep symptoms
- Home-based intervention, which reduces burden on participants
- Unable to blind participants to the treatment group
- Motor training component of the trial is optional and therefore will likely have a reduced sample size

For peer review only

For peer review only - http://bmjopen.bmj.com/site/about/guidelines.xhtml

Introduction

Stroke is one of the leading causes of long-term disability worldwide, often resulting in prolonged cognitive, motor, and emotional problems (1). Many stroke survivors also experience long-term sleep disruption (2-4), with the prevalence of post-stroke insomnia, a sleep disorder characterised by difficulties initiating and maintaining sleep, estimated to be as high as 60% (5-7). Poor sleep after stroke is frequently associated with motor/cognitive impairments, mood disorders such as anxiety and depression, and a reduced quality of life (8-10). There is also appreciable evidence to suggest that those who have poorer sleep show worse rehabilitation outcomes (8,11), which we hypothesise may be, at least in part, due to the importance of sleep for learning and memory processes.

Motor learning – the process of learning new, or relearning old, motor skills – is an integral part of post-stroke rehabilitation (12). Improvements in motor performance not only occur during practise itself, but also during periods of rest, including sleep (13). In recent years, a large body of evidence has emerged indicating these latter improvements in performance, better known as consolidation, are sleep-dependent (14-21). Mechanistically, the consolidation of motor learning during sleep is thought to occur via 'reactivations' of neural activity elicited during initial learning, with stronger reactivation correlating to a greater degree of memory consolidation (20, 22). It therefore stands to reason that consolidation of motor memories after or between therapy sessions may be impaired post-stroke, via direct disruption of these biological processes that occur during sleep, thus hindering recovery. Indeed, recent results from our lab have demonstrated that sleep disturbances during rehabilitation after acquired brain injury are associated with poorer motor recovery outcomes (8). Together these findings suggest that improving sleep quality post-stroke could

For peer review only - http://bmjopen.bmj.com/site/about/guidelines.xhtml

potentially also help to enhance rehabilitation outcomes. However, research and interventions focussed on improving symptoms of post-stroke insomnia are lacking.

Cognitive behavioural therapy (CBT) is a National Institute for Health and Care Excellence (NICE) recommended, first-line treatment for those with insomnia disorder (23-25), and is supported by a wealth of evidence for improving symptoms in otherwise healthy individuals (26, 27). Whereas few patients receive access to therapist-delivered CBT due to systemic barriers (28, 29), fully-automated digital CBT (dCBT) has the potential to provide access to evidence-based treatment at scale. We, and others, have shown that the delivery of dCBT can improve insomnia symptoms (30, 31), psychological well-being (32, 33), cognitive function (34), and is also feasible to use after stroke (35). Furthermore, preliminary data suggests that dCBT is also efficacious at improving self-reported measures of sleep and mood in adult, community dwelling, stroke survivors when compared to provision of sleep hygiene information (36).

Study Objectives

Given the possible benefit of improved sleep on recovery of motoric function after stroke, the overall aim of the present study is to explore the potential for sleep improvement, via dCBT, to impact on rehabilitation outcomes post-stroke. Termed INSPIRES-2 (improving sleep and learning in rehabilitation after stroke, part 2), we will more specifically aim to:

1. Test whether symptoms of insomnia are improved in stroke survivors following dCBT in comparison to treatment as usual.

For peer review only

2. Assess whether overnight consolidation of motor learning is improved after the delivery of dCBT in comparison to treatment as usual.
3. Determine whether actigraphy-derived sleep measures are improved after the delivery of dCBT in comparison to treatment as usual.
4. Identify if changes in sleep following dCBT correlate with changes in overnight consolidation of motor learning.
5. Test for changes in symptoms of depression and fatigue after dCBT in comparison with treatment as usual.
6. Explore the feasibility and potential effect size estimates of at-home motor training following dCBT or usual care.

Methods and Analysis

Participant Recruitment

Up to 100 participants with stroke affecting the upper limb will be recruited. Participants will be identified via: National Health Service (NHS) patient identification centres, from prior study involvement at the Wellcome Centre for integrative Neuroimaging (WIN) or Cognitive Neuropsychology Centre (University of Oxford), social media, stroke support groups, and word of mouth. Participants may be identified at any time following their stroke but will not be enrolled in the study until they have been discharged from inpatient care. There is no specific post-stroke period restriction for recruitment to this trial.

The trial opened to recruitment in September 2022, with a planned trial end date of December 2024. The study was registered on 22nd August 2022 (prior to recruitment of the first participant; NCT05511285)

For peer review only

Inclusion/Exclusion Criteria

Participants must be 18 years of age or above, discharged from inpatient care, and have a clinical diagnosis of stroke affecting the upper limb. Participants should, however, have sufficient movement to be able to perform the prescribed motor learning task. Individuals must be willing and able to give informed consent for participation in the study. Participants should also be interested in accessing a programme intentionally aimed at improving sleep quality and given the nature of the study, have reliable access to the internet. Individuals may not enter the study if any of the following apply: diagnosis of other neurological condition affecting movement (e.g., Parkinson's Disease) or an untreated diagnosed sleep disorder (e.g., sleep apnoea); experience uncontrolled seizures; have engaged in psychological therapy for insomnia in the past 12 months; are pregnant; or have planned inpatient admission (e.g., for rehabilitation) in the next 4 months that would impact on the ability to engage with the dCBT programme.

Sample Size Distribution

Based on prior work in our lab comparing dCBT with sleep hygiene information in stroke survivors (36) and other studies involving dCBT in people who suffer from insomnia, we estimate a medium between group effect size of $d = 0.7$. In order to achieve this, a sample size of $n = 76$ will be required ($\alpha = 0.05$, power = 80%, 2:1 random allocation ration dCBT:Control, two-tailed independent samples t-test). A sample size of $n = 100$ was selected to accommodate an approximate 25% dropout rate (determined from previous research experience). As the at-home motor training component of the trial is optional, we expect the resultant sample size to be reduced.

For peer review only - http://bmjopen.bmj.com/site/about/guidelines.xhtml

(Figure 1)

Trial Design

This study will be conducted as a randomised controlled trial with two parallel study arms: (1) digital cognitive behavioural therapy for insomnia (dCBT; experimental condition), and (2) treatment as usual (Treatment as usual; control condition), see Figure 1. The trial will therefore be dCBT + Treatment as usual vs Treatment as usual only and is reported in accordance with the Standard Protocol Items: Recommendations for Interventional Trials checklist (37) (see online supplementary file 1). Participants will receive a version of the participant information sheet and can ask the researchers any questions they may have. Electronic informed consent is obtained from the participant (using Jisc) before any study-specific procedures are performed (online supplementary file 2).

Baseline measures of sleep will be obtained via completion of the Sleep Condition Indicator (SCI: 38) and 7-night actigraphy recordings (measuring estimated total sleep time, wakefulness after sleep onset (WASO), and sleep fragmentation index). For the actigraphy, a waterproof monitor (MotionWatch 8, CamNTech) will be delivered to participants to be worn on their least affected wrist. Participants will also complete a sleep diary (paper or digital), detailing approximately what time they attempted sleep and woke up for each of the 7 nights. Mood and fatigue will be assessed using the Patient Health Questionnaire (PHQ-9: 39) and the Fatigue Severity Scale (FSS: 40, 41), respectively.

We will assess consolidation of motor learning using the commercial rehabilitation device; Gripable (Gripable Ltd). At home, participants will complete a learning task called Balloon Buddies™ (42) at specific times of day. The task will require

For peer review only - http://bmjopen.bmj.com/site/about/guidelines.xhtml

participants to squeeze and release the Gripable device (a hand grip dynamometer) with their affected hand in order to direct an avatar, which appears on an associated tablet, towards targets set along a predetermined path. In each attempt of the task, participants can score up to 50 points. The score participants achieve will be displayed on the screen at the end of each task attempt. We will randomly assign participants to complete one of three different 'levels' of the task, which only differ in regard to the target path but not in difficulty. Before starting the task, the Gripable will be calibrated to each participants' individual grip and release strength/ability. Participants will be instructed to first complete the task in the evening (between 8-10pm or at least 30 minutes prior to sleep). They will complete the task 10 times (recording how many targets they hit each time), lasting no longer than 30 mins. Pilot data in a previous sample of stroke survivors with upper limb impairment, indicates significant improvements in accuracy over 10 repetitions (Figure 2). They will then be asked to perform a retest the next morning (between 8-10am or at least 30 minutes after wake), completing the same task a further 5 times. Again, we will ask participants to record their scores and the retest should take less than 15 mins.

Age, sex, ethnicity, date of stroke/s, stroke symptom laterality, modified Rankin Scale (mRS), and current medications will also be collected. Additionally, we will use the STOP-Bang questionnaire (43) to assess the potential risk of obstructive sleep apnoea (OSA) and a further sleep disorder questionnaire (44) to screen for sleep conditions other than insomnia. All demographic information will be collected and stored using online questionnaires (Jisc) or over the phone depending on the participants' preference. Additionally, all equipment will either be sent to the participants' homes or delivered by a researcher (depending on location and

For peer review only

preference) and will be collected in person upon completion or return postage will be organised.

(Figure 2)

Randomisation and Blinding

Following baseline assessments, participants will be randomised at a 2:1 ratio (experimental:control) using an online randomisation software (rando.la), with minimisation of between group differences in age, sex, baseline SCI score and time since stroke (months). Once randomised, each participant will be assigned a sequentially numbered study ID. It is not possible to blind the researchers involved in the day-to-day running of the study or the participants to their intervention group. Analysis will be carried out by an individual not involved in day-to-day research activity and not explicitly informed of the experimental condition.

Intervention

For the intervention group, participants will be asked to continue any of their usual treatments. In addition to this, they will be provided with access to the commercially available dCBT programme, Sleepio (Big Health Ltd). The programme involves 6 sessions of automated dCBT, with a virtual professor, and access to a daily online sleep diary. dCBT sessions become available 7 days following completion of the previous session. Participants may also interact with an online community and library of resources. Participants will be provided with a web link to the programme and will need to sign up by entering their name, email address, and a predetermined participant ID. As part of the dCBT programme, participants will be required to

For peer review only

complete a daily online sleep diary for the duration of the programme and watch weekly videos pertaining to dCBT. Based on feedback from previous studies, they will also receive a booklet with additional details that may be useful for accessing the programme.

The control group will be asked to continue to adhere to their treatment as usual, with no further intervention measures specific to the research. The exact nature of this treatment will vary across participants. At post-intervention we will ask whether participants in the control group received any treatment for their sleep. Participants in the control group will be provided the opportunity to access the dCBT programme after completing the study, however, no data will be recorded from this.

Post-Intervention

Approximately 10 weeks following randomisation, assessments of sleep (SCI, 7-night actigraphy, sleep diary), mood (PHQ-9), fatigue (FSS), and motor consolidation (Gripable task) will be repeated. Participants will be randomly assigned a different level of motor learning task so that the movement pattern is different to that which they learnt at baseline. Any participants who have not completed the dCBT programme by 10 weeks (or do not wish to complete it) will still be given the opportunity to complete the post-intervention assessments. Once completed, any equipment will be picked up by a researcher or return postage organised (dependent on participant preference, location and desire to complete voluntary training, see next section).

Optional Motor Training

For peer review only

Before completion of the post-intervention assessments, all participants – regardless of group allocation – will be offered 4 weeks of home-based upper limb training. This training will involve daily use of the Gripable rehabilitation device and target a wide range of hand and arm movements. Participants will be asked to attempt to perform 1 hour of training per day, either in one session or broken up into shorter sessions throughout the day. Depending on their level of impairment, participants will be instructed by a researcher which games on the device will be most suitable and how to access them, but participants are free to explore the device as they wish. We will also ask participants to complete a training diary to record how many times they use the device each day and to estimate how long they spent practicing each day in total. Participants access the device through a research account, which allows the research team access to a dashboard of data. We will utilise the dashboard information to record the amount of time spent on each task and the number of movement repetitions performed each day. If participants decide to engage in this training, assessments of upper limb function will be conducted using the Action Research Arm Test (ARAT: 45), Box and Blocks Test (BBT: 46), The Nine-Hole Peg Test (9HPT: 47), and range of movement using the Gripable device (pronation/supination, wrist flexion/extension, radial/ulnar deviation and grip squeeze (strength) and release (%)). These assessments will be collected in-person, during the post-intervention visit when actigraphy and Gripable devices are delivered and repeated approximately 4 weeks later after completion of the training programme. Data from the optional training intervention will help to inform protocols and effect-size estimates of future studies combining sleep improvement and at-home rehabilitation programmes.

For peer review only - http://bmjopen.bmj.com/site/about/guidelines.xhtml

Outcome Measures

The primary outcome of this study will be self-reported insomnia symptoms pre to post intervention compared to treatment as usual, measured via SCI scores (38). SCI data will be collected during baseline assessments and approximately 10 weeks after randomisation, following either the dCBT intervention or treatment as usual. SCI has been shown to be a robust screening tool for the clinical evaluation of insomnia, with an estimated magnitude change of 7 scale points representing a meaningful improvement in insomnia symptoms (48). Higher values on the SCI indicate fewer symptoms of insomnia and a score of 16 or below suggests probable insomnia (38).

Secondary outcome measures include: change in overnight motor memory consolidation between groups; changes in actigraphy-derived sleep measures (estimated total sleep time, WASO, and sleep fragmentation index) between groups; correlations between changes in SCI and actigraphy-derived sleep measures and overnight motor memory consolidation in the dCBT group; and changes in symptoms of depression and fatigue between the dCBT and control groups. Overnight motor consolidation will be determined by changes in performance on the Gripable motor learning task from evening training to morning retest (49), measured at baseline and approximately 10 weeks following randomisation. Actigraphy-based sleep measures at baseline and approximately 10 weeks following randomisation will be extracted and analysed using custom software, MotionWare (CamNTech Ltd) and corroborated with sleep diary entries. The PHQ-9 and FSS, measured at baseline and approximately 10 weeks following randomisation, will be used to measure changes in depression and fatigue between the dCBT and control groups. In addition to these secondary outcomes, we intend to measure the adherence and efficacy of

For peer review only - http://bmjopen.bmj.com/site/about/guidelines.xhtml

at-home motor training during the optional intervention as an exploratory outcome. Training diaries and changes in upper limb function (ARAT, BBT and 9HPT scores) will help to determine feasibility and potential effect size estimates.

Data Analysis

Primary Outcome

The primary outcome, SCI score at post-intervention, will be analysed using intention-to-treat (ITT). An analysis of covariance (ANCOVA) will be used to test whether self-reported symptoms of insomnia are improved for the dCBT group in comparison to the control group. The model will test for differences in SCI scores between groups at the post-intervention time point (~10 weeks following randomisation), with baseline score, sex and time since stroke added as covariates. All ANCOVAs will be run in general linear model format.

Secondary/Exploratory Outcomes

Secondary outcomes will be tested using a complete case ITT population, including all participants for whom data is available at the post-intervention timepoint (irrespective of whether they completed the intervention or not). To test for changes in motor consolidation, we will determine the difference in performance from the end of evening training relative to the morning retest on the Gripable motor learning task at each time point. To determine if motor consolidation improves for the dCBT group compared to control, we will use an ANCOVA. The model will test for between group differences at post-intervention (~10 weeks following randomisation), with baseline consolidation score as a covariate. Changes in actigraphy-derived sleep measures will also be analysed using an ANCOVA, testing for differences between groups at

For peer review only

368 10 weeks following randomisation with baseline scores added as covariates. To
369 explore whether the magnitude of changes in sleep measures relate to changes in
370 motor consolidation, we will use correlational analysis (either Spearman's or
371 Pearson's correlations as appropriate), testing for associations between changes in
372 SCI and actigraphy-derived sleep measures (estimated total sleep time, WASO, and
373 sleep fragmentation index) with changes in motor consolidation. Changes in
374 symptoms of depression and fatigue in the dCBT group compared to the control
375 group will also be analysed using an ANCOVA to examine differences between
376 groups on the PHQ-9 and FSS at follow up, with baseline score added as a
377 covariate. For all analyses, we will include covariates such as age, sex, time since
378 stroke etc. as required.
379

380 **Patient and Public Involvement (PPI)**

381 A PPI contributor (RF) has been involved in the setup of the study, including
382 providing feedback on the dCBT programme and assisting in interviewing/appointing
383 the research assistant who will be responsible for day-to-day management of the
384 study. They have critically reviewed the protocol and will be included as a co-author
385 in all published material from this study.

386 Throughout our previous studies of sleep after stroke and brain injury, we have
387 discussed sleep concerns with patients in hospital and in the community (e.g., at
388 stroke user group meetings and stroke awareness day events). We have
389 encouraged patients to try-out and provide feedback on the motor learning task at
390 various public/patient engagement events before and throughout the design of the
391 study. We utilised this feedback to design the instructions we give to participants and
392 to decide on which level of difficulty would be appropriate. Stroke survivors have also

For peer review only

been involved in reviewing participant facing documents but will not be directly involved in recruitment of study participants or conduct of the study. We will seek further assistance from PPI contributors to help with preparation of lay summaries and dissemination of our results to ensure that these are meaningfully communicated to the stroke community.

Study Monitoring

Regular monitoring of participants will be performed by the research team as appropriate. Data will be evaluated for compliance with the protocol and accuracy in relation to source documents. The study may also be monitored or audited by responsible individuals from the University Sponsor and the NHS Trust(s).

At 10 weeks following randomisation we will check with participants for the potential occurrence of any adverse effects. Any adverse events (AEs) that occur during enrolment in the trial will be documented and participants will be advised on the best course of action. This may include withdrawing from certain assessments or contacting their General Practitioner (GP). Expected minor AEs include: mild skin irritation due to wearing the actigraphy monitor, tiredness/worsening of sleep as part of the sleep restriction included in the dCBT programme, minor distress caused by completing sleep and mood questionnaires etc.

There are no anticipated serious adverse events (SAEs) associated with this trial. Any serious adverse events (SAE) that occur will be reported to the appropriate research ethics committee (REC), where in the opinion of the Chief Investigator the event was ‘related’ (resulted from administration of any of the research procedures) and ‘unexpected’ in relation to those procedures. Reports of related and unexpected SAEs will be submitted within 15 working days of the Chief Investigator becoming

For peer review only - http://bmjopen.bmj.com/site/about/guidelines.xhtml

aware of the event, using the Health Research Authority (HRA) report of serious adverse event form (see HRA website).

Data Management

Access will be granted to authorised representatives from the Sponsor and host institution for monitoring and/or audit of the study. The participant will be referred to by their study ID, not by name, on all documents apart from the consent form. All trial data will be entered on paper and/or an electronic document (e.g., Microsoft Excel) and stored safely in confidential conditions.

Early Discontinuation/Withdrawal

During the trial a participant may choose to withdraw early at any time. In addition, an investigator may discontinue a participant from the trial if considered necessary for any reason including, but not limited to: ineligibility (either arising during the study or retrospectively having been overlooked at screening), significant protocol deviation*, s*ignificant non-compliance with study requirements, medical instability, and subsequent stroke affecting motor function or requiring readmission. The type of withdrawal and reason for withdrawal will be recorded. If permitted and appropriate, data obtained up until the point of withdrawal to be retained for use in the study analysis. No further data or samples would be collected after withdrawal. Withdrawn participants will be replaced providing there is sufficient time to conduct all follow up assessments within the study period.

Ethics and Dissemination

For peer review only - http://bmjopen.bmj.com/site/about/guidelines.xhtml

This study has received both Health Research Authority (HRA) and Health and Care Research Wales (HCRW) approval (IRAS ID: 306291). The study has also received approval from the National Research Ethics Service (22/EM/0080). The trial has been registered on a publicly accessible database prior to the recruitment of the first participant (clinicaltrials.gov NCT05511285). Results will be uploaded to clinicaltrials.gov within 12 months of the end of trial declaration by the CI or their delegate and disseminated via presentations at scientific conferences, peer-reviewed publication, public engagement events, stakeholder organisations, and other forms of media where appropriate. The investigators will be involved in reviewing drafts of the manuscripts, abstracts, press releases, and any other publications arising from the study.

For peer review only

Acknowledgements

We would like to thank the NIHR CRN Stroke for their support in setting up, recruiting for, and facilitating the trial.

Author Contributions

MKF conceived and designed the protocol with input from MW, BR, RF, CAE, SDK, ALH, BS, and HJB. EG, AP, RT, SA, and ND facilitate recruitment and data collection. MW and BR are involved in the day-to-day running of the trial. MW drafted the manuscript. All authors edited, revised and approved the final version of the manuscript.

Funding

This study is funded by Guarantors of Brain (Fellowship to MKF) and the Wellcome Trust (Principal Research Fellowship to HJB; 222446/Z/21/Z) and supported by the NIHR Oxford (Oxford University Hospitals) and Oxford Health Biomedical Research Centres (BRC). These funding sources had no role in the design of this study and will not have any role during its execution, analyses, or interpretation of data.

Competing Interests

CAE is co-founder and Chief Scientist of Big Health Ltd., is salaried and is a shareholder. ALH is employed by Big Health Ltd, is salaried and a shareholder. Big Health Ltd. are supplying Sleepio free of charge to the study.

Rights Retention

For peer review only

This research is funded in whole, or in part, by the Wellcome Trust [Grant number 222446/Z/21/Z]. For the purpose of open access, the author has applied a CCBY public copyright license to any Author Accepted Manuscript version arising from this submission.

For peer review only

References

(1) Feigin VL, Brainin M, Norrving B, Martins S, Sacco RL, Hacke W, et al. World Stroke Organization (WSO): global stroke fact sheet 2022. *International Journal of Stroke*. 2022;17(1):18-29. https://doi.org/10.1177/17474930211065917

(2) Duss SB, Seiler A, Schmidt MH, Pace M, Adamantidis A, Müri RM, et al. The role of sleep in recovery following ischemic stroke: a review of human and animal data. *Neurobiology of sleep and circadian rhythms*. 2017;2:94-105. https://doi.org/10.1016/j.nbscr.2016.11.003

(3) Sterr A, Kuhn M, Nissen C, Ettine D, Funk S, Feige B, et al. Post-stroke insomnia in community-dwelling patients with chronic motor stroke: Physiological evidence and implications for stroke care. *Scientific Reports*. 2018;8(1):1-9. doi: https://doi.org/10.1038/s41598-018-26630-y

(4) Khot SP, Morgenstern LB. Sleep and stroke. *Stroke*. 2019;50(6):1612-7. doi: https://doi.org/10.1161/STROKEAHA.118.023553

(5) Gottlieb E, Landau E, Baxter H, Werden E, Howard ME, Brodtmann A. The bidirectional impact of sleep and circadian rhythm dysfunction in human ischaemic stroke: a systematic review. *Sleep Medicine Reviews*. 2019;45:54-69. doi: https://doi.org/10.1016/j.smrv.2019.03.003

(6) Baylan S, Griffiths S, Grant N, Broomfield NM, Evans JJ, Gardani M. Incidence and prevalence of post-stroke insomnia: a systematic review and meta-analysis. *Sleep medicine reviews*. 2020;49:101222. https://doi.org/10.1016/j.smrv.2019.101222

(7) Hasan F, Gordon C, Wu D, Huang HC, Yuliana LT, Susatia B, et al. Dynamic prevalence of sleep disorders following stroke or transient ischemic attack: systematic review and meta-analysis. *Stroke*. 2021;52(2):655-63. https://doi.org/10.1161/STROKEAHA.120.029847

(8) Fleming MK, Smejka T, Henderson Slater D, van Gils V, Garratt E, Yilmaz Kara E, et al. Sleep disruption after brain injury is associated with worse motor outcomes and slower functional recovery. *Neurorehabilitation and Neural Repair*. 2020;34(7):661- 71. https://doi.org/10.1177/154596832092966

(9) Fulk GD, Boyne P, Hauger M, Ghosh R, Romano S, Thomas J, et al. The impact of sleep disorders on functional recovery and participation following stroke: A systematic review and meta-analysis. *Neurorehabilitation and Neural Repair*. 2020;34(11):1050-61. doi: https://doi.org/10.1177/1545968320962501

For peer review only

(10) Fleming MK, Smejka T, Henderson Slater D, Chiu EG, Demeyere N, Johansen-Berg H. Self-reported and objective sleep measures in stroke survivors with incomplete motor recovery at the chronic stage. *Neurorehabilitation and Neural Repair*. 2021;35(10):851-60. https://doi.org/10.1177/15459683211029889

(11) Iddagoda MT, Inderjeeth CA, Chan K, Raymond WD. Post-stroke sleep disturbances and rehabilitation outcomes: a prospective cohort study. *Internal Medicine Journal*. 2020;50(2):208-13. doi: https://doi.org/10.1111/imj.14372

(12) Krakauer JW. Motor learning: its relevance to stroke recovery and neurorehabilitation. *Current opinion in neurology*. 2006;19(1):84-90. doi: 10.1097/01.wco.0000200544.29915.cc

(13) Walker MP, Brakefield T, Seidman J, Morgan A, Hobson JA, Stickgold R. Sleep and the time course of motor skill learning. *Learning & memory*. 2003;10(4):275-84. doi: 10.1101/lm.58503

(14) Stickgold R, James L, Hobson JA. Visual discrimination learning requires sleep after training. *Nature neuroscience*. 2000;3(12):1237-8. doi: https://doi.org/10.1038/81756

(15) Stickgold R, Whidbee D, Schirmer B, Patel V, Hobson JA. Visual discrimination task improvement: A multi-step process occurring during sleep. *Journal of cognitive neuroscience*. 2000;12(2):246-54. https://doi.org/10.1162/089892900562075

(16) Fischer S, Hallschmid M, Elsner AL, Born J. Sleep forms memory for finger skills. *Proceedings of the National Academy of Sciences*. 2002;99(18):11987-91. doi: https://doi.org/10.1073/pnas.182178199

(17) Walker MP, Brakefield T, Morgan A, Hobson JA, Stickgold R. Practice with sleep makes perfect: sleep-dependent motor skill learning. *Neuron*. 2002;35(1):205-11. doi: https://doi.org/10.1016/S0896-6273(02)00746-8

(18) Huber R, Felice Ghilardi M, Massimini M, Tononi G. Local sleep and learning. *Nature*. 2004;430(6995):78-81. doi: https://doi.org/10.1038/nature02663

(19) Gudberg C, Wulff K, Johansen-Berg H. Sleep-dependent motor memory consolidation in older adults depends on task demands. *Neurobiology of aging*. 2015;36(3):1409- 16. doi: https://doi.org/10.1016/j.neurobiolaging.2014.12.014

(20) Ramanathan DS, Gulati T, Ganguly K. Sleep-dependent reactivation of ensembles in motor cortex promotes skill consolidation. *PLoS biology*. 2015;13(9):e1002263. doi: https://doi.org/10.1371/journal.pbio.1002263

For peer review only - http://bmjopen.bmj.com/site/about/guidelines.xhtml

(21) Belal S, Cousins J, El-Deredy W, Parkes L, Schneider J, Tsujimura H, et al. Identification of memory reactivation during sleep by EEG classification. *Neuroimage*. 2018;176:203-14. https://doi.org/10.1016/j.neuroimage.2018.04.029

(22) Deuker L, Olligs J, Fell J, Kranz TA, Mormann F, Montag C, et al. Memory consolidation by replay of stimulus-specific neural activity. *Journal of Neuroscience*. 2013;33(49):19373-83. https://doi.org/10.1523/JNEUROSCI.0414-13.2013

(23) Qaseem A, Kansagara D, Forciea MA, Cooke M, Denberg TD, of the American College of Physicians* CGC. Management of chronic insomnia disorder in adults: a clinical practice guideline from the American College of Physicians. *Annals of internal medicine*. 2016;165(2):125-33. https://doi.org/10.7326/M15-2175

(24) Riemann D, Baglioni C, Bassetti C, Bjorvatn B, Dolenc Groselj L, Ellis JG, et al. European guideline for the diagnosis and treatment of insomnia. *Journal of sleep research*. 2017;26(6):675-700. https://doi.org/10.1111/jsr.12594

(25) Edinger JD, Arnedt JT, Bertisch SM, Carney CE, Harrington JJ, Lichstein KL, et al. Behavioral and psychological treatments for chronic insomnia disorder in adults: an American Academy of Sleep Medicine clinical practice guideline. *Journal of Clinical Sleep Medicine*. 2021;17(2):255-62. https://doi.org/10.5664/jcsm.8986

(26) Trauer JM, Qian MY, Doyle JS, Rajaratnam SM, Cunnington D. Cognitive behavioral therapy for chronic insomnia: a systematic review and meta-analysis. *Annals of internal medicine*. 2015;163(3):191-204. doi: https://doi.org/10.7326/M14-2841

(27) Van Straten A, van der Zweerde T, Kleiboer A, Cuijpers P, Morin CM, Lancee J. Cognitive and behavioral therapies in the treatment of insomnia: a meta-analysis. *Sleep medicine reviews*. 2018;38:3-16. doi: https://doi.org/10.1016/j.smrv.2017.02.001

(28) Koffel E, Bramoweth AD, Ulmer CS. Increasing access to and utilization of cognitive behavioral therapy for insomnia (CBT-I): a narrative review. *Journal of General Internal Medicine*. 2018;33(6):955-62. https://doi.org/10.1007/s11606-018-4390-1

(29) Thomas A, Grandner M, Nowakowski S, Nesom G, Corbitt C, Perlis ML. Where are the behavioral sleep medicine providers and where are they needed? A geographic assessment. *Behavioral sleep medicine*. 2016;14(6):687-98. https://doi.org/10.1080/15402002.2016.1173551

(30) Espie CA, Kyle SD, Williams C, Ong JC, Douglas NJ, Hames P, et al. A randomized, placebo-controlled trial of online cognitive behavioral therapy for chronic

For peer review only

insomnia disorder delivered via an automated media-rich web application. *Sleep*. 2012;35(6):769-81. doi: https://doi.org/10.5665/sleep.1872

(31) Luik AI, Kyle SD, Espie CA. Digital cognitive behavioral therapy (dCBT) for insomnia: a state-of-the-science review. *Current Sleep Medicine Reports*. 2017;3(2):48-56. https://doi.org/10.1007/s40675-017-0065-4

(32) Espie CA, Emsley R, Kyle SD, Gordon C, Drake CL, Siriwardena AN, et al. Effect of digital cognitive behavioral therapy for insomnia on health, psychological well- being, and sleep-related quality of life: a randomized clinical trial. *JAMA psychiatry*. 2019;76(1):21-30. doi: 10.1001/jamapsychiatry.2018.2745

(33) Henry AL, Miller CB, Emsley R, Sheaves B, Freeman D, Luik AI, et al. Insomnia as a mediating therapeutic target for depressive symptoms: a sub-analysis of participant data from two large randomized controlled trials of a digital sleep intervention. *Journal of sleep research*. 2021;30(1):e13140. https://doi.org/10.1111/jsr.13140

(34) Kyle SD, Hurry ME, Emsley R, Marsden A, Omlin X, Juss A, et al. The effects of digital cognitive behavioral therapy for insomnia on cognitive function: a randomized controlled trial. *Sleep*. 2020;43(9):zsaa034. doi: https://doi.org/10.1093/sleep/zsaa034

(35) Smejka T, Henry AL, Wheatley C, Espie CA, Johansen-Berg H, Fleming MK. A qualitative examination of the usability of a digital cognitive behavioral therapy for insomnia program after stroke. *Brain Injury*. 2022:1-8. doi: https://doi.org/10.1080/02699052.2022.2034182

(36) Fleming MK, Smejka T, Macey E, Luengo-Fernandez R, Henry AL, Robinson B, Kyle SD, Espie CA, Johansen-Berg H. Improving sleep after stroke: a randomised controlled trial of digital cognitive behavioural therapy for insomnia. medRxiv. 2023:2023-02. https://doi.org/10.1101/2023.02.07.23285580

(37) Chan AW, Tetzlaff JM, Altman DG, Laupacis A, Gøtzsche PC, Krleža-Jeri´c K, et al. SPIRIT 2013 statement: defining standard protocol items for clinical trials. *Annals of internal medicine*. 2013;158(3):200-7. https://doi.org/10.7326/0003-4819-158-3-201302050-00583

(38) Espie CA, Kyle SD, Hames P, Gardani M, Fleming L, Cape J. The Sleep Condition Indicator: a clinical screening tool to evaluate insomnia disorder. *BMJ open*. 2014;4(3):e004183. doi: doi:10.1136/bmjopen-2013-004183

(39) Kroenke K, Spitzer RL, Williams JB. The PHQ-9: validity of a brief depression severity measure. *Journal of general internal medicine*. 2001;16(9):606-13. https://doi.org/10.1046/j.1525-1497.2001.016009606.x

For peer review only - http://bmjopen.bmj.com/site/about/guidelines.xhtml

(40) Schwartz JE, Jandorf L, Krupp LB. The measurement of fatigue: a new instrument. *Journal of psychosomatic research*. 1993;37(7):753-62. https://doi.org/10.1016/0022-3999(93)90104-N

(41) Lerdal A, Kottorp A. Psychometric properties of the Fatigue Severity Scale—Rasch analyses of individual responses in a Norwegian stroke cohort. *International journal of nursing studies*. 2011;48(10):1258-65. https://doi.org/10.1016/j.ijnurstu.2011.02.019

(42) Mace M, Kinany N, Rinne P, Rayner A, Bentley P, Burdet E. Balancing the playing field: collaborative gaming for physical training. *Journal of neuroengineering and rehabilitation*. 2017;14(1):1-18. https://doi.org/10.1186/s12984-017-0319-x

(43) Chung F, Abdullah HR, Liao P. STOP-Bang questionnaire: a practical approach to screen for obstructive sleep apnea. *Chest*. 2016;149(3):631-8. https://doi.org/10.1378/chest.15-0903

(44) Wilson SJ, Nutt D, Alford C, Argyropoulos S, Baldwin DS, Bateson A, et al. British Association for Psychopharmacology consensus statement on evidence-based treatment of insomnia, parasomnias and circadian rhythm disorders. *Journal of psychopharmacology*. 2010;24(11):1577-601. https://doi.org/10.1177/0269881110379307

(45) Lyle RC. A performance test for assessment of upper limb function in physical rehabilitation treatment and research. *International journal of rehabilitation research*. 1981;4(4):483-92.

(46) Mathiowetz V, Volland G, Kashman N, Weber K. Adult norms for the Box and Block Test of manual dexterity. *The American journal of occupational therapy*. 1985;39(6):386-91. doi: https://doi.org/10.5014/ajot.39.6.386

(47) Mathiowetz V, Weber K, Kashman N, Volland G. Adult norms for the nine hole peg test of finger dexterity. *The Occupational Therapy Journal of Research*. 1985;5(1):24- 38. doi: https://doi.org/10.1177/153944928500500102

(48) Espie CA, Farias Machado P, Carl JR, Kyle SD, Cape J, Siriwardena AN, et al. The Sleep Condition Indicator: reference values derived from a sample of 200 000 adults. *Journal of sleep research*. 2018;27(3):e12643. https://doi.org/10.1111/jsr.12643

(49) Robertson EM, Pascual-Leone A, Miall RC. Current concepts in procedural consolidation. *Nature Reviews Neuroscience*. 2004;5(7):576-82. https://doi.org/10.1038/nrn1426

For peer review only - http://bmjopen.bmj.com/site/about/guidelines.xhtml

Figure Legends

Figure 1: *Flow chart of the trial, depicting patient recruitment, screening, intervention, assessments, and optional motor training. Sleep Condition Indicator; SCI, Modified Rankin Scale; mRS, Obstructive Sleep Apnoea Questionnaire; STOP-Bang, Patient Health Questionnaire; PHQ-9, Fatigue Severity Scale; FSS, Digital Cognitive behavioural Therapy; dCBT, Action Research Arm Test; ARAT, Box and Blocks Test; BBT, Nine-Hole Peg Test; 9HPT.*

Figure 2: *Number of targets hit (maximum 50) per repetition of the Gripable motor learning task in a sample of stroke survivors with upper limb impairment. Mean scores indicated by filled circles ± standard error (sample size: n = 25).*

For peer review only

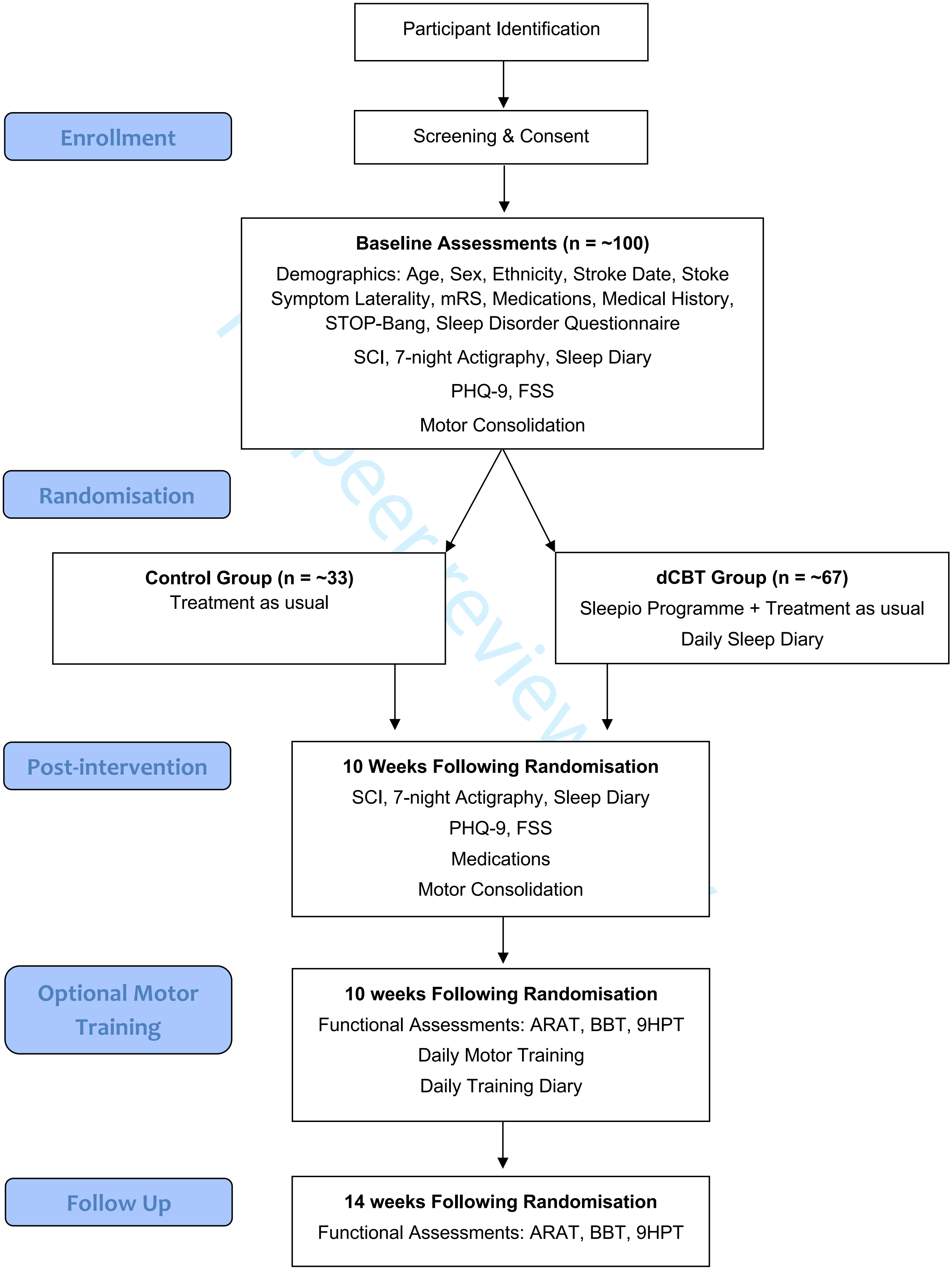
Participant Identification
Enrollment
Screening & Consent
Baseline Assessments (n = ~100)
Demographics: Age, Sex, Ethnicity, Stroke Date, Stoke Symptom Laterality, mRS, Medications, Medical History, STOP-Bang, Sleep Disorder Questionnaire
SCI, 7-night Actigraphy, Sleep Diary
PHQ-9, FSS
Motor Consolidation
Randomisation
Control Group (n = ~33)
Treatment as usual
dCBT Group (n = ~67)
Sleepio Programme + Treatment as usual
Daily Sleep Diary
Post-intervention
10 Weeks Following Randomisation
SCI, 7-night Actigraphy, Sleep Diary
PHQ-9, FSS
Medications
Motor Consolidation
Optional Motor Training
10 weeks Following Randomisation
Functional Assessments: ARAT, BBT, 9HPT
Daily Motor Training
Daily Training Diary
Follow Up
14 weeks Following Randomisation
Functional Assessments: ARAT, BBT, 9HPT

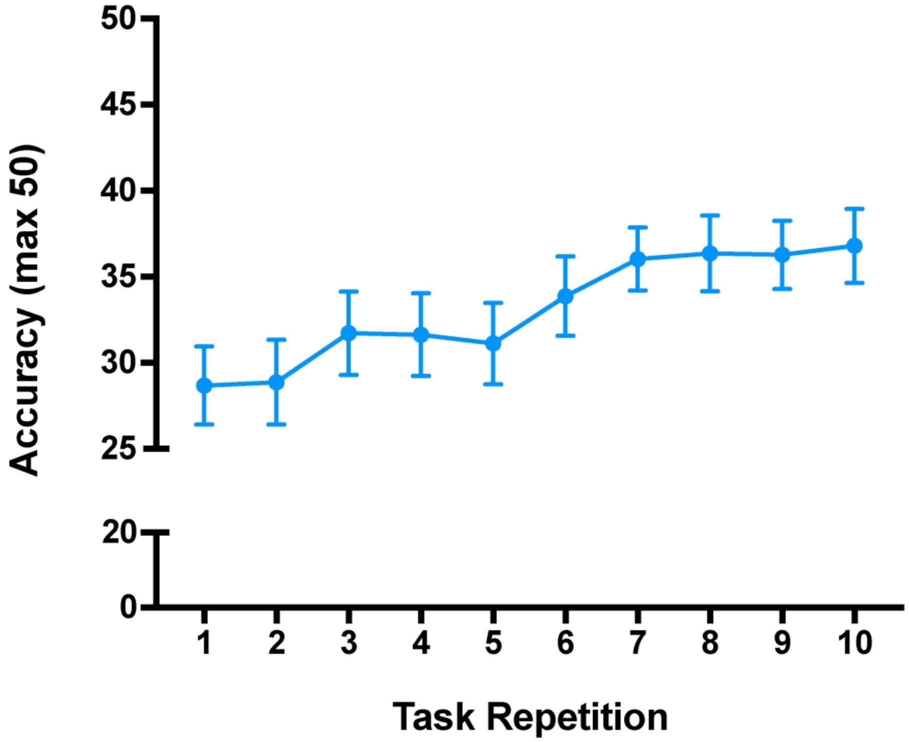

Number of targets hit (maximum 50) per repetition of the Gripable motor learning task in a sample of stroke survivors with upper limb impairment. Mean scores indicated by filled circles ± standard error (sample size: n = 25).

127x98mm (600 x 600 DPI)

Reporting checklist for protocol of a clinical trial.

Based on the SPIRIT guidelines.

Instructions to authors

Complete this checklist by entering the page numbers from your manuscript where readers will find each of the items listed below.

Your article may not currently address all the items on the checklist. Please modify your text to include the missing information. If you are certain that an item does not apply, please write "n/a" and provide a short explanation.

Upload your completed checklist as an extra file when you submit to a journal.

In your methods section, say that you used the SPIRITreporting guidelines, and cite them as:

Chan A-W, Tetzlaff JM, Gøtzsche PC, Altman DG, Mann H, Berlin J, Dickersin K, Hróbjartsson A, Schulz KF, Parulekar WR, Krleža-Jerić K, Laupacis A, Moher D. SPIRIT 2013 Explanation and Elaboration: Guidance for protocols of clinical trials. BMJ. 2013;346:e7586

		Reporting Item	Page Number
**Administrative information**			
Title	#1	Descriptive title identifying the study design, population, interventions, and, if applicable, trial acronym	1
Trial registration	#2a	Trial identifier and registry name. If not yet registered, name of intended registry	4
Trial registration: data set	#2b	All items from the World Health Organization Trial Registration Data Set	n/a
Protocol version	#3	Date and version identifier	4
Funding	#4	Sources and types of financial, material, and other support	26

For peer review only

Roles and responsibilities: contributorship	#5a	Names, affiliations, and roles of protocol contributors	1 & 26
Roles and responsibilities: sponsor contact information	#5b	Name and contact information for the trial sponsor	1
Roles and responsibilities: sponsor and funder	#5c	Role of study sponsor and funders, if any, in study design; collection, management, analysis, and interpretation of data; writing of the report; and the decision to submit the report for publication, including whether they will have ultimate authority over any of these activities	26
Roles and responsibilities: committees	#5d	Composition, roles, and responsibilities of the coordinating centre, steering committee, endpoint adjudication committee, data management team, and other individuals or groups overseeing the trial, if applicable (see Item 21a for data monitoring committee)	16-18 & 26

Introduction

Background and rationale	#6a	Description of research question and justification for undertaking the trial, including summary of relevant studies (published and unpublished) examining benefits and harms for each intervention	5-6
Background and rationale: choice of comparators	#6b	Explanation for choice of comparators	7
Objectives	#7	Specific objectives or hypotheses	6-7
Trial design	#8	Description of trial design including type of trial (eg, parallel group, crossover, factorial, single group),	8-10

For peer review only

		allocation ratio, and framework (eg, superiority, equivalence, non-inferiority, exploratory)	
**Methods: Participants, interventions, and outcomes**			
Study setting	#9	Description of study settings (eg, community clinic, academic hospital) and list of countries where data will be collected. Reference to where list of study sites can be obtained	9
Eligibility criteria	#10	Inclusion and exclusion criteria for participants. If applicable, eligibility criteria for study centres and individuals who will perform the interventions (eg, surgeons, psychotherapists)	7-8
Interventions: description	#11a	Interventions for each group with sufficient detail to allow replication, including how and when they will be administered	11-12
Interventions: modifications	#11b	Criteria for discontinuing or modifying allocated interventions for a given trial participant (eg, drug dose change in response to harms, participant request, or improving / worsening disease)	17-18
Interventions: adherance	#11c	Strategies to improve adherence to intervention protocols, and any procedures for monitoring adherence (eg, drug tablet return; laboratory tests)	16-18
Interventions: concomitant care	#11d	Relevant concomitant care and interventions that are permitted or prohibited during the trial	8 & 11-12
Outcomes	#12	Primary, secondary, and other outcomes, including the specific measurement variable (eg, systolic blood pressure), analysis metric (eg, change from baseline, final value, time to event), method of aggregation (eg, median, proportion), and time point for each outcome. Explanation of the clinical relevance of chosen efficacy and harm outcomes is strongly recommended	13-14

For peer review only

Participant timeline	#13	Time schedule of enrolment, interventions (including any run-ins and washouts), assessments, and visits for participants. A schematic diagram is highly recommended (see Figure)	Figure 1
Sample size	#14	Estimated number of participants needed to achieve study objectives and how it was determined, including clinical and statistical assumptions supporting any sample size calculations	8
Recruitment	#15	Strategies for achieving adequate participant enrolment to reach target sample size	8
**Methods: Assignment of interventions (for controlled trials)**			
Allocation: sequence generation	#16a	Method of generating the allocation sequence (eg, computer-generated random numbers), and list of any factors for stratification. To reduce predictability of a random sequence, details of any planned restriction (eg, blocking) should be provided in a separate document that is unavailable to those who enrol participants or assign interventions	12
Allocation concealment mechanism	#16b	Mechanism of implementing the allocation sequence (eg, central telephone; sequentially numbered, opaque, sealed envelopes), describing any steps to conceal the sequence until interventions are assigned	10-11
Allocation: implementation	#16c	Who will generate the allocation sequence, who will enrol participants, and who will assign participants to interventions	10-11

For peer review only

Blinding (masking)	#17a	Who will be blinded after assignment to interventions (eg, trial participants, care providers, outcome assessors, data analysts), and how	10-11
Blinding (masking): emergency unblinding	#17b	If blinded, circumstances under which unblinding is permissible, and procedure for revealing a participant's allocated intervention during the trial	n/a
**Methods: Data collection, management, and analysis**			
Data collection plan	#18a	Plans for assessment and collection of outcome, baseline, and other trial data, including any related processes to promote data quality (eg, duplicate measurements, training of assessors) and a description of study instruments (eg, questionnaires, laboratory tests) along with their reliability and validity, if known. Reference to where data collection forms can be found, if not in the protocol	8-10, 12-13
Data collection plan: retention	#18b	Plans to promote participant retention and complete follow-up, including list of any outcome data to be collected for participants who discontinue or deviate from intervention protocols	16-17
Data management	#19	Plans for data entry, coding, security, and storage, including any related processes to promote data quality (eg, double data entry; range checks for data values). Reference to where details of data management procedures can be found, if not in the protocol	17
Statistics: outcomes	#20a	Statistical methods for analysing primary and secondary outcomes. Reference to where other details of the statistical analysis plan can be found, if not in the protocol	14-15

For peer review only

Statistics: additional analyses	#20b	Methods for any additional analyses (eg, subgroup and adjusted analyses)	14-15
Statistics: analysis population and missing data	#20c	Definition of analysis population relating to protocol non-adherence (eg, as randomised analysis), and any statistical methods to handle missing data (eg, multiple imputation)	n/a
**Methods: Monitoring**			
Data monitoring: formal committee	#21a	Composition of data monitoring committee (DMC); summary of its role and reporting structure; statement of whether it is independent from the sponsor and competing interests; and reference to where further details about its charter can be found, if not in the protocol. Alternatively, an explanation of why a DMC is not needed	n/a
Data monitoring: interim analysis	#21b	Description of any interim analyses and stopping guidelines, including who will have access to these interim results and make the final decision to terminate the trial	17-18
Harms	#22	Plans for collecting, assessing, reporting, and managing solicited and spontaneously reported adverse events and other unintended effects of trial interventions or trial conduct	16-17
Auditing	#23	Frequency and procedures for auditing trial conduct, if any, and whether the process will be independent from investigators and the sponsor	16-18
**Ethics and dissemination**			
Research ethics approval	#24	Plans for seeking research ethics committee / institutional review board (REC / IRB) approval	3

For peer review only

Protocol amendments	#25	Plans for communicating important protocol modifications (eg, changes to eligibility criteria, outcomes, analyses) to relevant parties (eg, investigators, REC / IRBs, trial participants, trial registries, journals, regulators)	26-27
Consent or assent	#26a	Who will obtain informed consent or assent from potential trial participants or authorised surrogates, and how (see Item 32)	8-10
Consent or assent: ancillary studies	#26b	Additional consent provisions for collection and use of participant data and biological specimens in ancillary studies, if applicable	n/a
Confidentiality	#27	How personal information about potential and enrolled participants will be collected, shared, and maintained in order to protect confidentiality before, during, and after the trial	11, 17-18
Declaration of interests	#28	Financial and other competing interests for principal investigators for the overall trial and each study site	26
Data access	#29	Statement of who will have access to the final trial dataset, and disclosure of contractual agreements that limit such access for investigators	17
Ancillary and post trial care	#30	Provisions, if any, for ancillary and post-trial care, and for compensation to those who suffer harm from trial participation	n/a
Dissemination policy: trial results	#31a	Plans for investigators and sponsor to communicate trial results to participants, healthcare professionals, the public, and other relevant groups (eg, via publication, reporting in results databases, or other data sharing arrangements), including any publication restrictions	18
Dissemination policy: authorship	#31b	Authorship eligibility guidelines and any intended use of professional writers	n/a

For peer review only

Dissemination policy: reproducible research	#31c	Plans, if any, for granting public access to the full protocol, participant-level dataset, and statistical code	26-27

Appendices

Informed consent materials	#32	Model consent form and other related documentation given to participants and authorised surrogates	Supplementary file 2
Biological specimens	#33	Plans for collection, laboratory evaluation, and storage of biological specimens for genetic or molecular analysis in the current trial and for future use in ancillary studies, if applicable	n/a

None The SPIRIT Explanation and Elaboration paper is distributed under the terms of the Creative Commons Attribution License CC-BY-NC. This checklist can be completed online using https://www.goodreports.org/, a tool made by the EQUATOR Network in collaboration with Penelope.ai

For peer review only

Dr Melanie Fleming
Sleep-win@ndcn.ox.ac.uk

Participant identification number: [ ][ ][ ][ ][ ][ ][ ]

CONSENT FORM

Study Title: INSPIRES-2

If you agree, please initial box

1. I confirm that I have read the information sheet dated................... (version............) for this study. I have had the opportunity to consider the information, ask questions and have had these answered satisfactorily.		
2. I understand that my participation is voluntary and that I am free to withdraw at any time without giving any reason, without my medical care or legal rights being affected.		
3. I understand that relevant sections of my medical notes and data collected during the study may be looked at by individuals from University of Oxford, from regulatory authorities and from the NHS Trust(s), where it is relevant to my taking part in this research. I give permission for these individuals to have access to my records.		
4. I agree to take part in this study.		
Additional:		
5. *I agree to be contacted about ethically approved research studies for which I may be suitable. I understand that agreeing to be contacted does not oblige me to participate in any further studies.*	Yes	No
6. *I would like the researchers to send a letter to my GP to let them know that I am taking part in this research (if yes, please give details)*	Yes	No

For peer review only - http://bmjopen.bmj.com/site/about/guidelines.xhtml

Name of Participant	*Date*

When completed: 1 for participant; 1 for researcher

If you agreed for researchers to send a letter to your GP, please provide your GP details

GP name (if known):

Surgery name:

Surgery address:

For peer review only

For peer review only - http://bmjopen.bmj.com/site/about/guidelines.xhtml
